# Cost–effectiveness analysis of revised WHO guidelines for management of childhood pneumonia in 74 Countdown countries

**DOI:** 10.7189/jogh.07.010409

**Published:** 2017-06

**Authors:** Shanshan Zhang, Beatrice Incardona, Shamim A Qazi, Karin Stenberg, Harry Campbell, Harish Nair, Severe ALRI Working Group

**Affiliations:** 1 Usher Institute of Population Health Sciences and Informatics, University of Edinburgh, Edinburgh, UK; 2 Department of Preventive Dentistry, Peking University School and Hospital of Stomatology, Beijing, China; 3 Università Cattolica del Sacro Cuore, Milan, Italy; 4 Department of Maternal, Newborn, Child and Adolescent Health, World Health Organization, Geneva, Switzerland; 5 Department of Health Systems Governance and Financing, World Health Organization, Geneva, Switzerland; 6 Public Health Foundation of India, New Delhi, India; * Membership of the Severe ALRI Working Group is available in the Acknowledgements section.

## Abstract

**Background:**

Treatment of childhood pneumonia is a key priority in low–income countries, with substantial resource implications. WHO revised their guidelines for the management of childhood pneumonia in 2013. We estimated and compared the resource requirements, total direct medical cost and cost-effectiveness of childhood pneumonia management in 74 countries with high burden of child mortality (Countdown countries) using the 2005 and 2013 revised WHO guidelines.

**Methods:**

We constructed a cost model using a bottom up approach to estimate the cost of childhood pneumonia management using the 2005 and 2013 WHO guidelines from a public provider perspective in 74 Countdown countries. The cost of pneumonia treatment was estimated, by country, for year 2013, including costs of medicines and service delivery at three different management levels. We also assessed country–specific lives saved and disability adjusted life years (DALYs) averted due to pneumonia treated in children aged below five years. The cost-effectiveness of pneumonia treatment was estimated in terms of cost per DALY averted by fully implementing WHO treatment guidelines relative to no treatment intervention for pneumonia.

**Results:**

Achieving full treatment coverage with the 2005 WHO guidelines was estimated to cost US$ 2.9 (1.9–4.2) billion compared to an estimated US$ 1.8 (0.8–3.0) billion for the revised 2013 WHO guidelines in these countries. Pneumonia management in young children following WHO treatment guidelines could save up to 39.8 million DALYs compared to a zero coverage scenario in the year 2013 in the 74 Countdown countries. The median cost-effectiveness ratio per DALY averted in 74 countries was substantially lower for the 2013 guidelines: US$ 26.6 (interquartile range IQR: 17.7–45.9) vs US$ 38.3 (IQR: US$ 26.2–86.9) per DALY averted for the 2005 guideline respectively.

**Conclusions:**

Child pneumonia management as detailed in standard WHO guidelines is a very cost–effective intervention. Implementation of the 2013 WHO guidelines is expected to result in a 39.5% reduction in treatment costs compared to the 2005 guidelines which could save up to US$ 1.16 (0.68–1.23) billion in the 74 Countdown countries, with potential savings greatest in low HIV burden countries which can implement effective community case management of pneumonia.

Globally, pneumonia is a leading cause of mortality and morbidity in young children accounting for about 0.935 million deaths in 2013 (15% of under–five mortality) [1] and 120 million episodes worldwide in 2010 [2]. Implementation of the World Health Organization (WHO) and United Nations International Children's Emergency Fund (UNICEF) recommended integrated management of childhood illness (IMCI) strategy can potentially reduce 32 to 70% of pneumonia–specific under–five mortality [3–5]. However, treatment of childhood pneumonia places a large economic burden on families and the health care system, especially in resource–constrained countries. Severe acute lower respiratory infections (ALRI) place a substantial burden on health services worldwide and is a major cause of hospital referral and admission in young children [6]. The World Health Report (WHR) 2005 estimated that if a comprehensive package of child survival interventions was scaled up to 95% coverage in 74 high (child mortality) burden countries [7], by 2015 the costs for managing pneumonia would be equivalent to an *additional* US$ 1.48 per capita (2004 US$, inflated to 1.83 in 2013 US$) from public provider’s perspective.

In 2013, WHO revised the guidelines for the management of childhood pneumonia [8–10]. The key change in the 2013 guidelines is that Human Immunodeficiency Virus (HIV)–uninfected children (aged 1 month – 4 years) with lower chest wall in–drawing (with or without tachypnoea) are classified as having pneumonia and recommended management at first level facilities (as out–patients) with oral dispersible amoxicillin instead of co–trimoxazole and no longer need to be treated at a hospital (see Box S1 in **Online Supplementary Document(Online Supplementary Document)**). They also recommend that HIV–infected children with lower chest wall in–drawing should be considered as having severe pneumonia and referred for hospital admission and treatment with ampicillin plus gentamicin IM or IV. Several clinical trials in the past few years have suggested that the treatment protocols in the revised (2013) guidelines are as effective as those in the previous (2005) guidelines in terms of measured clinically–defined rates of treatment failure and from this it has been inferred that they should have at least a similar impact on mortality [11–18].

However, we are not aware of any studies to date that report country level cost estimates for the 2013 WHO treatment guidelines. Existing studies that report cost of treatment of pneumonia mainly focus on the cost of illness per episode for each individual patient and demonstrate a considerable degree of methodological heterogeneity [19]. This greatly limits the extent to which valid national and international economic analyses can be carried out to inform international child health policy on pneumonia.

We aimed to estimate the cost of pediatric pneumonia management (from a public provider perspective) using the 2013 WHO guidelines, estimate cost-effectiveness (and cost savings) compared to use of the 2005 guidelines and estimate the country–specific annual investment required for childhood pneumonia management in the 74 Countdown countries prioritised by the “Countdown to 2015” initiative. These countries account for 97% of the maternal and child deaths worldwide [7].

## METHODS

### Study design

We constructed a cost model using a bottom up approach to estimate the total cost of pneumonia management using the 2005 and 2013 WHO guidelines from a public provider perspective in the 74 Countdown countries. In the absence of information on the clients’ preferred use of providers and challenges related to making predictions on private/public split, we applied public provider cost profiles to the analysis. We also used the WHO recommended template [20] to assess country–specific lives saved and disability adjusted life years (DALYs) averted in pneumonia cases (in children aged below five years receiving treatment in the 74 Countdown countries (comparing universal treatment coverage of cases to no treatment). DALYs for a disease were calculated as the sum of the Years of Life Lost (YLL) due to premature mortality in the population and the Years Lost due to Disability (YLD) for people living with the disease condition and its consequences. These 74 priority countries account for a population of 5.1 billion, with 520 million children aged below five years in 2013, and 97% of global pneumonia deaths [21].

We estimated the total cost of pneumonia treatment at a country level in the year 2013 assuming universal coverage (100%), including the total cost for medicine and service delivery at three different levels – community, first level health facility, and first referral hospital level. The total medicine cost and service delivery cost in each country were calculated for each delivery level based on the coverage and population in need. We used the standard treatment protocol recommended by the WHO including recommended dosage for antibiotics, supportive therapy and duration of treatment [8]. We estimated the cost of management of pneumonia in HIV–infected and uninfected children following the 2005 and 2013 WHO guidelines. In the absence of empirical data, we assumed that the proportion of pneumonia signs (ie, fast breathing, lower chest wall in–drawing and danger signs) are the same in HIV–infected and uninfected children with pneumonia – 85% of children with pneumonia have fast breathing, 13% have chest wall in–drawing and about 2% have danger signs – based on the results of studies carried out at the community level [15,22,23].

### Cost model inputs

The 74 countries were categorised into four groups based on HIV prevalence and the presence or absence of an implemented pneumonia community case management (CCM) policy. Community case management refers to an integrated strategy to achieve high treatment coverage and delivering high–quality care to sick children in the community.by community health workers. A country was defined as “high HIV prevalence country” when the adult prevalence (15–49 years) was above 1% in 2012. The proportion of the adult population infected by HIV was obtained from UNAIDS (2012) [24]. The standard treatment procedure in children who are HIV positive and negative are different as per the WHO guidelines. Information on policy and implementation of CCM were obtained from the Countdown Reports for 2014 [25] and 2012 [7]; these influenced assumptions regarding the proportion of the rural population seeking treatment at first level facility or community level.

The estimated country–specific population in need for each level of intervention was calculated using three parameters: population size, pneumonia incidence and urban/rural distribution of the population. The exposed population in need was calculated using the country level population of children aged below five years from the UN World Population Prospects [21], and the most recent published country–specific estimates of incidence of pneumonia among children below five years [26]. The population living in rural areas was estimated using the UN World Urbanisation Prospects (2011) [27], and we estimated the population in need at the community and facility level for urban and rural areas separately. The number of pneumonia cases in HIV–infected children was obtained from recently published estimates [26].

We assumed universal coverage of pneumonia treatment with 100% children affected by pneumonia being treated either at community, first level health facility or first referral hospital level. Community level treatment implies a community health worker (CHW) treating the child at home. In countries with implementation of a CCM policy, we assumed that half of the rural population would be treated by CHWs and the remaining half would be treated at a health facility [15,19]. We assumed that the urban population would be treated at first level health facility and first referral hospital level. We conducted sensitivity analyses to examine the change in the overall cost of pneumonia treatment by varying the coverage of the rural population by CHWs. We considered two different types of direct medical costs – cost of medicines and costs related to service delivery. All costs are presented in US dollars (2013), and are estimated by level of intervention and country. We used median supplier prices from Management Sciences for Health (MSH) International Price Indicator (2012) [28], UNICEF supply division data, and UNICEF report for cost of medicines [29] for the list of drugs based on the previous and revised WHO guidelines. Costs were estimated for an average child weighing 10 kg (around one–year–old) due to lack of age disaggregated population data.

Country–specific service delivery costs (ie, costs for one inpatient day and one outpatient visit) were obtained from the World Health Organization Choosing Interventions that are Cost–Effective project (WHO–CHOICE) estimates [30] (which include operational costs such as health worker consultation time, electricity and maintenance of health facility buildings). We applied outpatient unit costs from WHO–CHOICE for service delivery at the first level facility and inpatient unit costs for service delivery at the first referral level hospital. For the community level service delivery, we used the salary received by CHWs in the published literature [31], which was supplemented by consultation with experts from WHO. The number of CHWs needed per country was obtained by assuming one CHW per 1000 rural population [31].

### Effectiveness of pneumonia treatment

The effectiveness of pneumonia treatment was measured in terms of country–specific disability adjusted life years (DALYs) averted by implementing WHO treatment guidelines relative to no treatment intervention for pneumonia, ie, implementation of 2005 guidelines vs no treatment and implementation of 2013 guidelines vs no treatment. Country–specific DALYs were computed using the WHO DALY Calculation Template [20,32]. We obtained country–specific population data by gender for children aged 0–4 years and the life expectancy at birth for both the sexes from the World Population Prospect (2013) [21], and used pneumonia specific incidence and mortality estimates published recently [1,2,26]. Deaths averted by pneumonia treatment were calculated based on the reported estimates of 70% child mortality reduction from universal coverage of community management of childhood pneumonia [33]. We assumed that the 2005 and 2013 guidelines have the same treatment effectiveness and that community management of fast breathing pneumonia and health facility management of fast breathing and lower chest in–drawing pneumonia with oral amoxicillin (based on the clinical trials [11–18]) have similar effectiveness in terms of mortality reduction. We assumed that the peak incidence of childhood pneumonia was at 1.5 months and that median age at death was 8.9 months based on unpublished data from Kilifi, Kenya, (Jay Berkley, personal communication); these were also supported by published literature and expert opinion [34,35]. Disability weight is a weighting factor that reflects the severity of the disease on a scale from 0 (perfect health) to 1 (dead). For our analysis, we applied a standard disability weight of 0.21 for infectious disease (acute episode, severe) [36].

Cost-effectiveness is measured in terms of cost per DALY averted in each country. The thresholds for considering an intervention to be cost–effective were set following the recommendations of the Commission on Macroeconomics and Health [37]. Interventions that cost less than three times the average gross domestic product (GDP) per capita income per DALY averted were considered to be cost–effective and an intervention whose cost per DALY averted was less than the average per capita income for a given country was considered to be highly cost–effective.

### Sensitivity analysis

We simulated the cost estimates using several scenarios by varying unit cost, length of stay in hospital, level of coverage and population in need (Box S2 in **Online Supplementary Document(Online Supplementary Document)**). We extracted an actual cost data scenario derived from a systematic review of the published literatures based on 34 cost studies, and incorporated these data into our model to compare the results with the standard prices model [19]. We also conducted sensitivity analyses assuming 100% rural coverage for CCM implementation, 36% of mortality reduction, 1 CHW per 5000 rural population and 9%–30% of effective access rate to health care in rural areas [38]. Additionally, we performed several analyses to examine a range of total costs and cost savings in each country to assess whether / within what parameter settings the intervention remained cost–effective.

We conducted the analyses using Microsoft Excel 2010 (Microsoft Corp., Redmond, WA, USA).

## RESULTS

### Total cost and cost savings

**Figure 1:**
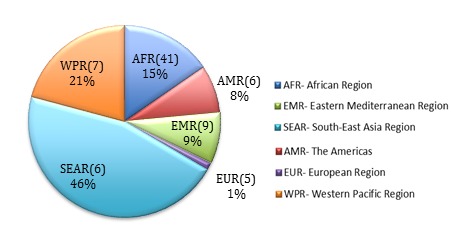
Percentage of Total Cost Savings by WHO Regions (number of countries in each region), total estimated cost savings in 2013: US$ 1.16 billion. Total cost savings (Unit: Billion) SEAR: 0.53; WPR: 0.24; AFR: 0.18; EMR: 0.099; AMR: 0.094; EUR: 0.014.

The total direct medical cost of management of all–cause childhood pneumonia in the 74 Countdown countries in 2013 using the 2005 IMCI guidelines was estimated to be about US$ 2.94 billion. However, implementation of the 2013 IMCI guidelines would cost approximately US$ 1.78 billion (**Table 1**). Thus, a total of US$ 1.16 billion could potentially be saved by implementing the 2013 guidelines in these countries. About 82% of these cost savings could be achieved in countries in the WHO South–East Asia, Africa and Western Pacific regions (**Figure 1**), which account for the majority of cases and deaths due to childhood pneumonia. These findings can be further broken down into cost at the three delivery levels – pneumonia management at community and first level facilities cost more per child with pneumonia when following the 2013 guidelines, but this was outweighed by the substantial reduction in costs at the hospital level (**Table 2**).

**Table 1:** Total cost of management of childhood pneumonia in 74 Countdown countries in 2013

**Table 2:** Total treatment cost by country for each level of service delivery*

Our estimates of country–specific cost for the management of childhood pneumonia demonstrate that ten countries (Bangladesh, China, India, Mexico, Pakistan, Indonesia, Philippines, Brazil, Nigeria and South Africa) account for about three quarters of the total cost in the 74 Countdown countries (**Table 3**). Potentially up to US$ 933 million could be saved in these ten countries alone by fully implementing the 2013 guidelines which corresponds to about 80% of the total cost savings in the 74 countries.

**Table 3:** Total cost of management of childhood pneumonia by HIV status

### Cost savings by HIV prevalence

In low HIV prevalence countries, treatment costs for HIV–infected patients accounted for less than one percent of the total cost of pneumonia management. Countries with high HIV prevalence require a larger share (between 0.82% and 65.20%) of the total direct medical cost investment to manage pneumonia in young HIV–infected children. Following implementation of the 2013 guidelines, countries implementing CCM can achieve substantial cost savings of US$ 1.00 billion collectively compared to savings of only $0.16 billion in those not implementing CCM, particularly in low HIV burden settings (Table S1 in **Online Supplementary Document(Online Supplementary Document)**).

### Total cost per capita

Globally, the total cost per capita for direct medical pneumonia costs of treatment (based on an assumed 100% treatment coverage) ranged from US$ 0.04 (in Brazil on one extreme) to US$ 2.13 (in Equatorial Guinea at the other extreme). Similarly, total health care expenditure per capita using the 2013 guidelines ranged from 0.0035% (Brazil on the lower end) to 12.47% (Somalia on the higher end) (**Table 2**). The median total cost per capita for the 2013 guidelines among 74 countries was US$ 0.38 (IQR: US$ 0.24–0.47), which represented 0.52% (IQR: 0.15%–1.14%) of total health care expenditure per capita. This is lower than that for the 2005 guideline – US$ 0.54 (IQR: US$ 0.40–0.75). In seven countries (Eritrea, Niger, Somalia, Burundi, Central African Republic, Madagascar, and Ethiopia), the estimated total cost of pneumonia treatment per capita exceeds 2% of the total health care expenditure per capita following the 2013 guidelines.

### Cost–effectiveness of 2005 and 2013 guidelines

Assuming that the two sets of guidelines are equally effective (see above) then adopting the 2013 guidelines should help many countries achieve a more cost–effective pneumonia management strategy and should result in substantial savings per DALY averted, compared to the 2005 guidelines. Following the parameters described above and 100% treatment coverage, our model estimates that up to 39.8 million DALYs could be averted in the 74 countries. We also estimate that the total Years of Life Saved would be 38.8 million and total deaths averted would be about 0.63 million. This yields a median cost–effectiveness ratio of $38.3 (IQR: US$ 26.2–86.9) and US$ 26.6 (IQR: 17.7–45.9) per DALY averted using the 2005 and 2013 guidelines respectively. The median cost per DALY averted for 74 countries was US$ 11.5 (IQR US$ 7.2–30.0). We estimated that nine countries with highest cost per DALYS averted – China, Mexico, Peru, Brazil, Gabon, Botswana, Equatorial Guinea, Azerbaijan and South Africa – could save between US$ 95.6 and US$ 284.8 per DALY averted if the 2013 guidelines were fully implemented (**Table 4**).

**Table 4:** Total DALYs averted and cost–effectiveness of implementing 2013 guidelines

### Sensitivity analysis results

We conducted sensitivity analyses by varying the unit price of medicine (Tables S2–S4 in **Online Supplementary Document(Online Supplementary Document)**), CHW/rural population ratio and CHW coverage (Tables S5–S7 in **Online Supplementary Document(Online Supplementary Document)**), effective access to health care in rural areas (Tables S8–S10 in **Online Supplementary Document(Online Supplementary Document)**) and the proportion of pneumonia signs in HIV– infected children with pneumonia (Tables S11–S13 in **Online Supplementary Document(Online Supplementary Document)**, higher cost from published studies but less effective scenario (Tables S14–S18 in **Online Supplementary Document(Online Supplementary Document)**). This way we designed 6 likely scenarios (See Box S2 in **Online Supplementary Document(Online Supplementary Document)**). These analyses demonstrated that the total direct medical cost of pneumonia treatment was US$ 1.9–4.2 billion based on universal coverage of the 2005 WHO guidelines and US$ 0.8–3.0 billion following the 2013 guidelines. The estimated cost savings (from a public provider perspective) by implementing the 2013 guidelines ranged between US$ 0.7 and US$ 1.2 billion, eg, in the effective access scenario, with a 9%–30% effective access rate applied to rural population, total cost saving was US$ 0.78 billion (Table S8 in **Online Supplementary Document(Online Supplementary Document)**). The median cost–effectiveness ratio ranged from US$ 12.8 (IQR: 8.8–25.6) to US$ 56.0 (34.7–120.2) per DALY averted. Both 2005 and 2013 guidelines for the pneumonia treatment interventions remained highly cost–effective in all scenarios.

### Discussion

Treatment of childhood pneumonia poses a substantial economic burden in resource limited low–income countries. Our study is the first to estimate the direct medical cost to the public sector of pneumonia management at community, first level facility, and first referral hospital levels using the 2005 and 2013 WHO guidelines. In this analysis we have studied “pneumonia” as defined by WHO case management and realize that this case definition encompasses other acute lower respiratory conditions such as bronchioliitis. We have demonstrated that implementation of the 2013 IMCI guidelines for the treatment of childhood pneumonia is expected to result in substantial cost savings (up to 39.5% of the budget for pneumonia treatment) and saved 38.8 millions years of life globally (Table S19 in **Online Supplementary Document(Online Supplementary Document)**). More than 80% of the estimated global savings would be made by implementing the 2013 guidelines in ten high burden countries alone (**Table 2**). Although implementation of these guidelines will result in slightly higher costs (per patient) at community and first level facility levels due to the use of oral amoxicillin (**Table 2**), the substantial reduction in costs at the hospital level would substantially decrease the overall economic burden on the health care system in low–income countries (**Table 2**) and save monies. However, the cost savings are comparatively lower in high HIV–burden settings (**Table 3**).

This study shows the significant financial and social benefits that would accrue and the substantial reduction in burden on inpatient hospital services that would result from full implementation of the 2013 WHO guidelines. The difference in direct medical costs of pneumonia management is mainly due to the reduction in costs at the hospital level, because most of the pneumonia cases with lower chest in–drawing will NOT be admitted but will be treated on an outpatient basis at the first level facility. Implementing the revised guidelines will thus result in reduction in hospitalization that will reduce the burden on already overcrowded and poorly resourced hospitals. Fewer beds will be utilized by these (less severely ill) children so benefiting more severely ill children, with pneumonia or other severe illness, for whom those beds (and consequent care from in–patient hospital staff) will become available. There will also result in less pressure on over–stretched inpatient staff, limited resources (such as oxygen which can then be targeted better at children with hypoxaemia), a reduced risk of hospital infections and fewer injection related serious adverse events. In addition, the reduction in hospitalization rates can be expected to reduce non–medical costs and the social and financial burden on families by avoiding family costs associated with hospitalisation. Implementing the 2013 WHO guidelines will also make effective treatment with less expensive medicines more locally available to families nearer their homes. This will act to reduce transport costs, loss of wages and other opportunity costs.

Although WHO and UNICEF issued joint statements supporting community case management of pneumonia, diarrhea (and later malaria) in 2004 [39], CCM as an integrated package was introduced only in 2012 [40]. Despite there being clear scientific evidence and an established global consensus regarding benefits of integrated community services for childhood illnesses, the uptake of CCM has been limited. Presently, integrated CCM guidelines are being implemented in only 47 of the 74 Countdown countries [25]. The potential savings in the annual recurrent cost by implementing the 2013 WHO guidelines (as demonstrated in this paper), could be used to support the costs in starting–up the CCM strategy and/or scaling–up the coverage of CCM.

Our estimates have several limitations. First, due to lack of empirical evidence, we have assumed that the proportion of children with pneumonia who have tachypnoea and those who have lower chest wall in–drawing and / danger signs remains same in HIV negative and HIV positive children. This is unlikely to be the case, as children with HIV who are not on anti–retroviral therapy, are more likely to develop (very) severe disease and die. Preliminary large–scale data from Malawi (high HIV burden with integrated CCM implementation) indicates that in program settings, as many as 13% of the children with pneumonia have been reported by CHW to have danger signs compared to 2% in our model (Tim Colbourn, personal communication). In the model, treatment procedures and cost implications for HIV positive patients with chest in–drawing and danger signs are the same in 2005 and 2013 guidelines (Box S1 in **Online Supplementary Document(Online Supplementary Document)**). Thus as long as the total percentage of patients with chest in–drawing and danger signs remains the same, the proportion of the subgroups does not influence the total management cost. While this does not alter the total direct medical costs much in our study, with regards to implementation of the 2013 IMCI guidelines, this does indicate the need for further research on community–based case ascertainment of pneumonia in high HIV burden settings. Moreover, in this study, we estimated the cost of childhood pneumonia caused by viruses or bacteria, and did not include complications of pneumonia, such as pleural effusion and empyema, lung abscess and pneumothorax. Children presenting other conditions with wheeze (such as bronchiolitis and asthma), with stridor (such as viral croup or diphtheria) or chronic cough (such as tuberculosis and pertussis) were not included.

Second, we have assumed that in integrated CCM implemented settings, 50% of the rural population would be covered by CHWs and the number of CHWs needed per country was obtained by assuming one CHW per 1000 rural population. Available data indicate that this may be difficult to achieve. Therefore, we conducted sensitivity analysis assuming a coverage among rural populations of 9% (when no CCM is implemented), and 30% when CCM is fully implemented (Tables S8–10 in **Online Supplementary Document(Online Supplementary Document)**). We also considered a scenario with one CHW per 5000 rural population. We found cost savings in all these scenarios (Tables S11–13 in **Online Supplementary Document(Online Supplementary Document)**). In the extreme scenario with 100% of the rural population were treated by community health workers the results remain cost–effective (Tables S2–S4 in **Online Supplementary Document(Online Supplementary Document)**).

Third, we only measured “one–off” direct medical cost from a health care provider’s perspective. Direct non–medical costs, such as transportation, over the counter medicines, food for patients and accompanying family members, and other out–of–pocket expenses were not considered in this model. Indirect costs (productivity loss and opportunity costs for caregivers) were also not included in this analysis. The recurrent costs and program costs of introducing a new policy in a country were not estimated here. These costs can often be substantial.

Fourth, we applied a public provider cost profile only. Recent surveillance data in Guatemala, Kenya and Thailand found that private physicians treated up to 36% of severe respiratory illness [41]. However, the cost information for private care is difficult to obtain and estimate in these countries. By considering only a public provider profile our estimated costs are likely to under–estimate true resource needs, especially in settings in which private providers are more commonly consulted for treatment of pneumonia.

Fifth, we assumed the same clinical effectiveness for 2005 and 2013 guidelines (ie, they achieved 70% mortality reduction). However, there is some debate as to whether the 2013 guidelines can achieve the same effectiveness as the previous one [42]. We undertook a sensitivity analysis varying the clinical effectiveness estimate and found that the 2013 guidelines remained cost-effective when the mortality reduction from pneumonia management for 2013 guideline was only 36% (ie, roughly half that when following 2005 guidelines) (Table S16 in **Online Supplementary Document(Online Supplementary Document)**).

Our research demonstrates that implementing the WHO 2013 guidelines for pneumonia management is not only cost–effective, but can also generate substantial cost–savings for each country (compared to current costs implementing 2005 WHO guidelines). Estimation of these avoidable costs is highly relevant and should be of great interest to policy makers in developing countries and donor agencies. Our estimates provide a vital piece of evidence from an economic perspective, to encourage policy makers at the national level and external funding bodies to make informed decisions in setting priorities and budget lines for pneumonia treatment within national programmes. Additionally, the use of scarce resources could be maximized toward the development and advancement of integrated CCM where referral is not possible and improving the quality of care at the primary, secondary and tertiary level health facilities. We also postulate that the cost savings are mainly in low HIV settings. Therefore, further research is required into more cost-effective treatment strategies for pneumonia in high HIV burden settings, especially where there is good coverage with anti–retroviral therapy.

## 

**Table:** 

Delivery level	Total cost for pneumonia treatment in 2013 (Billions, in 2013 US$)				
**2005 Guidelines [****10****]**		**2013 Guidelines [****8****]**		**2013 Guidelines/2005 Guidelines %**
**Total Cost**	**% Total**	**Total cost**	**% Total**	
Community	1.25	42.4	1.25	70.4	100.3
First level facility	0.18	6.1	0.23	12.8	126.0
Hospital	1.51	51.4	0.30	16.9	19.9
Total cost	2.94	100	1.78	100.0	60.5

**Table:** 

Country	Total treatment cost by delivery level for 2005 Guidelines [10] (thousands, 2013 US$)			Total treatment cost by delivery level for 2013 Guideline [8] (thousands, 2013 US$)			Total treatment cost ratio: 2013 Guidelines/2005 Guidelines (%)			
**Community**	**First level facility**	**Primary hospital**	**Community**	**First level facility**	**Primary hospital**	**Community**	**First level facility**	**Primary hospital**	**Total**
Afghanistan	11 401.08	10 460.50	12 278.82	11 516.61	13 283.25	1794.78	101.01	126.98	14.62	77.90
Angola	–	1114.05	23 277.54	–	1401.03	5088.23	–	125.76	21.86	26.60
Azerbaijan	2120.28	1286.70	6289.06	2125.15	1561.02	1274.37	100.23	121.32	20.26	51.16
Bangladesh	54 523.08	2917.34	27 298.93	54 727.21	4065.44	4227.04	100.37	139.35	15.48	74.37
Benin	2764.68	128.71	2029.47	2775.73	190.67	358.01	100.40	148.13	17.64	67.53
Bolivia (Plurinational State of)	–	242.95	1709.18	–	297.57	316.86	–	122.48	18.54	31.47
Botswana	–	44.29	2430.51	–	57.19	766.37	–	129.11	31.53	33.28
Brazil	–	2295.24	26 878.76	–	2888.23	5004.98	–	125.84	18.62	27.06
Burkina Faso	6066.40	964.40	5864.39	6116.28	1301.36	981.56	100.82	134.94	16.74	65.13
Burundi	4399.71	709.60	1123.25	4414.11	923.06	199.84	100.33	130.08	17.79	88.84
Cambodia	–	455.79	1666.49	–	559.87	288.41	–	122.83	17.31	39.97
Cameroon	5182.60	258.02	5652.74	5202.16	381.90	1298.54	100.38	148.01	22.97	62.04
Central African Republic	–	6789.37	1118.01	–	7794.68	267.34	–	114.81	23.91	101.96
Chad	–	720.47	4500.48	–	926.15	941.21	–	128.55	20.91	35.77
China	332 126.14	4231.84	250 139.39	332 389.24	6056.05	48 630.31	100.08	143.11	19.44	66.00
Comoros	–	448.69	213.26	–	522.36	33.25	–	116.42	15.59	83.94
Congo	786.08	127.92	2897.28	789.56	169.37	699.90	100.44	132.40	24.16	43.52
Congo, Democratic Republic	21 593.58	1991.78	11 753.02	21 710.58	2730.74	1876.43	100.54	137.10	15.97	74.47
Côte d'Ivoire	–	2526.81	7 952.09	–	3 043.90	1 740.60	–	120.46	21.89	45.66
Djibouti	–	40.93	266.28	–	51.46	53.53	–	125.74	20.10	34.18
Egypt	–	1466.52	13,334.03	–	1809.30	2479.44	–	123.37	18.59	28.98
Equatorial Guinea	–	14.07	5042.05	–	19.36	1591.47	–	137.59	31.56	31.86
Eritrea	2421.25	85.68	2007.63	2429.03	122.15	380.68	100.32	142.57	18.96	64.94
Ethiopia	37 952.87	919.68	10 635.81	38 069.35	1359.98	1948.09	100.31	147.87	18.32	83.58
Gabon	–	49.79	2770.42	–	63.20	688.00	–	126.95	24.83	26.64
Gambia	384.33	87.02	388.63	386.11	111.45	70.00	100.46	128.08	18.01	66.00
Ghana	6053.11	350.63	4385.54	6067.41	474.13	866.40	100.24	135.22	19.76	68.66
Guatemala	3774.98	234.93	1857.80	3786.51	324.04	284.08	100.31	137.93	15.29	74.90
Guinea	3689.35	699.79	2357.00	3707.74	901.87	419.99	100.50	128.88	17.82	74.56
Guinea–Bissau	–	68.07	377.07	–	87.36	88.04	–	128.34	23.35	39.40
Haiti	–	517.71	1298.83	–	626.76	238.43	–	121.06	18.36	47.63
India	418 239.33	16 406.87	591 463.73	419 709.33	23 682.25	112 428.79	100.35	144.34	19.01	54.17
Indonesia	59 799.61	4929.74	35 865.47	59 913.35	6346.30	6344.94	100.19	128.73	17.69	72.18
Iraq	–	9823.46	20 550.40	–	11 456.60	4154.23	–	116.62	20.21	51.40
Kenya	–	5337.83	7865.87	–	6275.86	1904.43	–	117.57	24.21	61.95
Korea, Democratic People's Republic	4795.55	145.40	4851.59	4802.07	204.49	912.74	100.14	140.64	18.81	60.45
Kyrgyzstan	1743.10	99.15	1107.97	1748.98	136.66	183.49	100.34	137.83	16.56	70.13
Lao People's Democratic Republic	2164.27	117.75	1909.82	2173.38	167.33	332.71	100.42	142.10	17.42	63.78
Lesotho	–	50.08	472.47	–	63.35	218.85	–	126.49	46.32	54.00
Liberia	1561.98	261.60	660.00	1566.95	332.13	101.53	100.32	126.96	15.38	80.55
Madagascar	7520.89	944.39	4473.18	7559.02	1253.21	687.17	100.51	132.70	15.36	73.42
Malawi	6705.06	732.96	1979.92	6724.64	934.94	669.24	100.29	127.56	33.80	88.44
Mali	4848.14	1647.53	4249.70	4878.90	2094.76	722.41	100.63	127.15	17.00	71.62
Mauritania	1107.71	478.22	1172.87	1113.42	597.55	207.50	100.51	124.95	17.69	69.54
Mexico	12 994.34	1491.12	77 172.09	13 012.59	1921.17	15 409.53	100.14	128.84	19.97	33.11
Morocco	–	4556.94	6606.32	–	5309.92	1237.23	–	116.52	18.73	58.65
Mozambique	8652.85	5520.45	6414.29	8700.73	6780.13	1999.95	100.55	122.82	31.18	84.91
Myanmar	17 424.61	762.94	7406.96	17 467.28	1041.27	1272.04	100.24	136.48	17.17	77.28
Nepal	11 210.34	678.15	3103.69	11 239.79	907.35	477.82	100.26	133.80	15.40	84.21
Niger	7138.68	2330.37	4913.61	7193.46	3 030.11	741.45	100.77	130.03	15.09	76.24
Nigeria	42 534.99	26 108.57	59 338.83	42 732.87	31 949.59	13 508.53	100.47	122.37	22.77	68.91
Pakistan	56 508.00	16 699.70	46 987.80	56 741.27	21 059.19	7879.04	100.41	126.11	16.77	71.28
Papua New Guinea	3114.48	188.58	1986.34	3124.71	256.22	370.36	100.33	135.87	18.65	70.92
Peru	3352.26	124.13	9306.02	3356.20	181.53	1840.11	100.12	146.24	19.77	42.07
Philippines	24 463.96	3722.85	34 784.32	24 547.02	4779.18	6335.22	100.34	128.37	18.21	56.63
Rwanda	4627.50	308.74	1178.31	4638.29	405.88	224.79	100.23	131.46	19.08	86.17
Sao Tome and Principe	–	30.83	33.66	–	35.97	6.74	–	116.68	20.02	66.23
Senegal	3948.39	488.16	3059.40	3962.94	635.97	536.93	100.37	130.28	17.55	68.51
Sierra Leone	1801.09	248.21	1131.77	1810.32	327.56	189.91	100.51	131.97	16.78	73.18
Solomon Islands	–	78.99	126.27	–	92.97	22.40	–	117.69	17.74	56.20
Somalia	3185.18	1113.81	2841.88	3211.74	1431.19	410.91	100.83	128.50	14.46	70.77
South Africa	–	1275.60	49 053.75	–	1590.69	17 881.83	–	124.70	36.45	38.69
Sudan	–	11 281.13	22 475.25	–	13 405.65	3844.41	–	118.83	17.11	51.10
Swaziland	–	60.05	520.30	–	70.48	252.13	–	117.35	48.46	55.59
Tajikistan	2932.36	774.56	1777.77	2944.10	986.64	291.31	100.40	127.38	16.39	76.98
Tanzania, United Republic of	–	8832.22	7093.35	–	10 288.06	1676.30	–	116.48	23.63	75.13
Togo	2054.78	285.33	1198.07	2063.27	368.76	260.12	100.41	129.24	21.71	76.09
Turkmenistan	1305.42	85.85	2913.80	1308.94	115.77	566.89	100.27	134.85	19.46	46.26
Uganda	15 426.85	943.94	6083.92	15 480.64	1280.18	1522.94	100.35	135.62	25.03	81.43
Uzbekistan	8969.37	862.48	6421.00	8994.75	1121.98	1147.19	100.28	130.09	17.87	69.30
Viet Nam	–	4275.11	14 077.75	–	5150.35	2476.74	–	120.47	17.59	41.56
Yemen	8042.85	2660.84	11 772.38	8094.02	3398.63	2058.14	100.64	127.73	17.48	60.29
Zambia	4299.30	574.52	3296.80	4309.87	721.09	1172.36	100.25	125.51	35.56	75.92
Zimbabwe	–	643.02	2062.99	–	765.63	832.50	–	119.07	40.35	59.06
Total	1 247 712.75	180 231.49	1 511 555.44	1 251 337.59	227 059.41	300 210.04	100.42	125.98	19.86	60.51

*Total treatment cost ratio of Revised Guidelines vs Old guideline by treatment settings.

**Table:** 

Country	2005 Guidelines [10] (thousands, 2013 US$)			2013 Guidelines [8] (thousands, 2013 US$)			Total cost savings (thousands, 2013 US$)	2013 Guidelines	
**HIV+**	**HIV–**	**Total**	**HIV+**	**HIV–**	**Total**	**Total cost per capita**	**Proportion of national health care expenditure per Capita (%)**
Afghanistan	4.78	34 135.63	34 140.41	4.95	26 589.69	26 594.64	7545.77	0.87	1.56
Angola	588.79	23 802.80	24 391.59	808.42	5767.72	6576.13	17815.46	0.30	0.16
Azerbaijan	39.30	9656.73	9696.03	53.31	4907.24	4960.54	4735.49	0.53	0.15
Bangladesh	3.03	84 736.32	84 739.35	3.30	63 016.38	63 019.68	21 719.66	0.40	1.52
Benin	43.96	4878.91	4922.87	48.47	3275.94	3324.41	1598.46	0.32	0.88
Bolivia (Plurinational State of)	4.06	1948.07	1952.13	5.10	577.56	582.66	1369.47	0.06	0.05
Botswana	270.49	2204.32	2474.81	388.96	480.84	869.80	1605.01	0.41	0.09
Brazil	15.10	29 158.90	29 174.00	19.46	7847.54	7867.01	21 306.99	0.04	0.0035
Burkina Faso	132.04	12 763.14	12 895.19	140.12	8259.09	8399.21	4495.98	0.50	1.33
Burundi	75.95	6156.61	6232.56	76.33	5460.68	5537.02	695.55	0.54	2.33
Cambodia	24.87	2097.41	2122.28	27.60	754.28	781.88	1340.40	0.06	0.11
Cameroon	355.52	10 737.84	11 093.36	416.34	6466.27	6882.61	4210.75	0.31	0.45
Central African Republic	538.84	7368.55	7907.39	544.55	7521.47	8066.02	–158.64	1.75	9.55
Chad	289.80	4931.14	5220.94	318.28	1563.37	1881.65	3339.29	0.15	0.41
China	83.52	586 413.86	586 497.37	116.07	386 959.53	387 075.60	199 421.77	0.28	0.10
Comoros	0.25	661.70	661.95	0.26	487.15	487.41	174.54	0.76	1.78
Congo	140.61	3670.67	3811.28	185.79	1473.04	1658.82	2152.46	0.37	0.43
Congo, Democratic Republic	429.87	34 908.51	35 338.38	428.49	25 889.25	26 317.74	9020.64	0.39	1.98
Côte d'Ivoire	565.27	9913.63	10 478.90	637.47	4180.08	4817.55	5661.34	0.24	0.30
Djibouti	10.07	297.13	307.20	11.72	94.00	105.72	201.48	0.12	0.11
Egypt	1.05	14 799.49	14 800.54	1.35	4072.57	4073.92	10 726.63	0.05	0.04
Equatorial Guinea	514.83	4541.29	5056.12	776.24	935.46	1711.70	3344.42	2.13	0.17
Eritrea	17.15	4497.40	4514.55	21.54	2910.33	2931.87	1582.68	0.46	3.33
Ethiopia	552.14	48 956.23	49 508.37	567.44	40 809.98	41 377.42	8130.95	0.44	2.65
Gabon	127.20	2693.01	2820.21	185.24	588.53	773.77	2046.44	0.45	0.13
Gambia	13.10	846.88	859.98	14.09	553.47	567.55	292.43	0.31	1.12
Ghana	118.24	10 671.04	10 789.28	140.10	7267.84	7407.95	3381.33	0.29	0.38
Guatemala	16.60	5851.12	5867.72	17.41	4377.21	4394.63	1473.09	0.28	0.13
Guinea	99.32	6646.82	6746.14	103.90	4925.69	5029.59	1716.55	0.43	1.44
Guinea–Bissau	40.40	404.74	445.14	42.93	133.95	176.88	268.26	0.10	0.28
Haiti	54.97	1761.58	1816.55	58.94	808.36	867.31	949.24	0.08	0.15
India	1669.57	1 024 440.35	1 026 109.93	2193.12	553 627.25	555 820.37	470 289.56	0.44	0.75
Indonesia	125.39	100 469.43	100 594.82	149.35	72 455.25	72 604.59	27 990.23	0.29	0.31
Iraq	310.48	30 063.38	30 373.86	387.99	13 745.27	14 133.26	16 240.60	0.46	0.14
Kenya	1064.89	12 138.81	13 203.70	1146.41	7074.75	8221.16	4982.54	0.18	0.51
Korea, Democratic People's Republic	0.21	9792.33	9792.54	0.27	5919.03	5919.30	3873.24	0.24	0.63
Kyrgyzstan	0.43	2949.80	2950.22	0.49	2068.64	2069.13	881.09	0.37	0.52
Lao People's Democratic Republic	8.92	4182.93	4191.84	10.47	2662.95	2673.42	1518.42	0.39	1.07
Lesotho	168.05	354.51	522.56	190.83	101.93	292.76	229.79	0.14	0.10
Liberia	17.86	2465.72	2483.58	17.99	1982.63	2000.62	482.96	0.32	0.59
Madagascar	54.14	12 884.32	12 938.46	56.38	9443.03	9499.41	3439.05	0.41	2.18
Malawi	543.94	8873.99	9417.94	556.83	7771.98	8328.82	1089.12	0.51	1.65
Mali	108.31	10 637.06	10 745.37	115.13	7580.94	7696.07	3049.30	0.50	1.13
Mauritania	18.69	2740.12	2758.81	20.91	1897.56	1918.47	840.35	0.49	0.85
Mexico	44.11	91 613.44	91 657.55	63.93	30 279.36	30 343.29	61 314.27	0.25	0.04
Morocco	6.27	11 156.99	11 163.26	7.55	5847.48	5855.04	5308.22	0.20	0.11
Mozambique	1774.32	18 813.27	20 587.58	1841.68	15 639.12	17 480.80	3106.78	0.68	1.92
Myanmar	67.27	25 527.24	25 594.51	76.30	19 704.29	19 780.59	5813.92	0.37	1.65
Nepal	15.35	14 976.83	14 992.18	16.34	12 608.62	12 624.96	2367.23	0.45	1.38
Niger	80.04	14 302.62	14 382.67	81.83	10 883.19	10 965.02	3417.64	0.61	3.06
Nigeria	4233.83	123 748.56	127 982.39	4875.27	83 315.73	88 190.99	39 791.39	0.51	0.64
Pakistan	21.97	120 173.52	120 195.50	24.95	85 654.55	85 679.50	34 516.00	0.47	1.58
Papua New Guinea	20.99	5268.41	5289.40	25.47	3725.83	3751.30	1538.11	0.51	0.65
Peru	19.10	12763.31	12 782.41	26.85	5350.99	5377.84	7404.57	0.18	0.06
Philippines	3.61	62 967.52	62 971.13	4.54	35 656.88	35 661.42	27 309.71	0.36	0.38
Rwanda	62.14	6052.42	6114.56	65.85	5203.11	5268.96	845.59	0.45	0.71
Sao Tome and Principe	1.68	62.80	64.49	1.89	40.91	42.80	21.69	0.22	0.19
Senegal	37.10	7458.85	7495.95	42.18	5093.66	5135.84	2360.11	0.36	0.54
Sierra Leone	41.06	3140.00	3181.06	42.06	2285.72	2327.79	853.27	0.38	0.56
Solomon Islands	0.83	204.44	205.27	0.95	102.53	103.48	101.79	0.21	0.15
Somalia	47.85	7093.02	7140.86	47.93	5005.91	5053.84	2087.03	0.48	12.47
South Africa	7868.39	42 460.96	50 329.35	11230.10	9556.77	20 786.88	29 542.47	0.37	0.05
Sudan	126.39	33 629.99	33 756.38	142.23	17 114.97	17 257.20	16 499.17	0.45	0.44
Swaziland	169.06	411.29	580.35	219.61	123.37	342.98	237.37	0.26	0.10
Tajikistan	5.36	5479.34	5484.70	5.92	4216.14	4222.05	1262.65	0.51	0.95
Tanzania, United Republic of	1243.91	14 681.66	15 925.57	1287.95	10 703.65	11 991.59	3933.98	0.24	0.65
Togo	105.00	3433.18	3538.18	110.67	2581.48	2692.15	846.03	0.39	0.88
Turkmenistan	4.00	4301.07	4305.08	5.50	1986.10	1991.60	2313.48	0.38	0.29
Uganda	788.10	21 666.61	22 454.70	816.27	17 467.48	18 283.75	4170.95	0.49	1.15
Uzbekistan	26.54	16 226.31	16 552.84	31.81	11232.11	11 263.92	4988.92	0.39	0.44
Viet Nam	74.25	18 278.60	18 352.85	86.23	6906.74	6992.97	11 359.88	0.08	0.09
Yemen	14.65	22 461.41	22 476.07	17.31	13 533.47	13 550.78	8925.28	0.56	0.63
Zambia	690.95	7479.67	8170.62	797.46	5405.86	6203.32	1967.29	0.43	0.49
Zimbabwe	709.98	1996.04	2706.02	760.05	865.22	1625.27	1080.74	0.11	0.29
Total	27 566.10	2 911 933.58	2 939 499.68	33767.00	1 743 376.97	1 777 143.97	1 162 355.70		

**Table:** 

Country	Total DALY averted (thousands)	Cost per DALY averted (2013 US$)				Percentage of cost per DALY averted in GDP per capita (%)	
**2005 Guidelines [****10****]**		**2013 Guidelines [****8****]**	**2005 Guidelines**		**2013 Guidelines**
Afghanistan	875.44	39.00	30.38		6.29		4.90
Angola	971.25	25.11	6.68		0.46		0.12
Azerbaijan	45.64	212.44	108.68		2.87		1.47
Bangladesh	910.09	93.11	69.25		12.46		9.27
Benin	191.87	25.66	17.33		3.41		2.30
Bolivia (Plurinational State of)	81.97	23.81	7.50		0.92		0.29
Botswana	9.75	253.83	84.47		3.53		1.17
Brazil	167.92	173.73	47.00		1.53		0.41
Burkina Faso	390.63	33.01	21.50		5.20		3.39
Burundi	250.15	24.92	22.14		9.93		8.82
Cambodia	120.25	17.65	7.05		1.87		0.75
Cameroon	499.15	22.22	13.79		1.93		1.20
Central African Republic	113.76	69.51	70.87		14.71		14.99
Chad	516.30	10.11	3.62		1.14		0.41
China	1752.85	334.60	220.83		5.41		3.57
Comoros	13.03	50.80	42.64		6.12		5.13
Congo	37.44	101.79	44.30		3.23		1.40
Congo, Democratic Republic	1770.31	19.96	14.87		7.34		5.47
Côte d'Ivoire	391.80	26.75	12.21		2.15		0.98
Djibouti	11.01	27.90	9.54		2.63		0.90
Egypt	227.57	65.04	18.85		2.04		0.59
Equatorial Guinea	14.30	353.62	112.66		1.47		0.47
Eritrea	98.98	45.61	29.62		9.04		5.87
Ethiopia	1568.40	31.57	26.38		6.71		5.61
Gabon	16.33	172.73	46.01		1.51		0.40
Gambia	29.69	28.96	19.11		5.66		3.73
Ghana	352.92	30.57	20.99		1.90		1.31
Guatemala	132.69	44.22	33.12		1.32		0.99
Guinea	224.05	30.11	22.45		5.09		3.80
Guinea–Bissau	41.79	10.65	4.20		1.97		0.78
Haiti	172.73	10.52	5.01		1.36		0.65
India	8522.64	120.40	65.22		8.08		4.38
Indonesia	1137.93	88.40	63.80		2.49		1.79
Iraq	265.05	114.60	58.90		1.78		0.91
Kenya	817.99	16.14	10.00		1.87		1.16
Korea, Democratic People's Republic	82.09	119.29	72.11		23.58		14.25
Kyrgyzstan	24.97	118.16	82.87		10.19		7.15
Lao People's Democratic Republic	124.61	33.64	21.46		2.40		1.53
Lesotho	24.95	20.94	11.31		1.76		0.95
Liberia	64.60	38.45	30.97		9.12		7.34
Madagascar	370.93	34.88	25.61		7.80		5.72
Malawi	215.99	43.60	38.56		16.27		14.39
Mali	515.92	20.83	14.92		3.00		2.15
Mauritania	80.87	34.11	23.72		3.08		2.14
Mexico	212.37	431.59	142.88		4.43		1.47
Morocco	151.78	73.55	43.13		2.53		1.49
Mozambique	423.41	48.62	41.29		8.40		7.13
Myanmar	327.07	78.25	60.48		6.84		5.29
Nepal	165.78	90.43	76.15		12.80		10.78
Niger	635.69	22.63	17.25		5.91		4.51
Nigeria	4499.06	28.45	19.60		1.83		1.26
Pakistan	3212.46	37.42	26.67		2.90		2.07
Papua New Guinea	96.55	54.79	38.86		2.51		1.78
Peru	57.94	220.62	92.82		3.36		1.41
Philippines	595.98	105.66	59.84		4.08		2.31
Rwanda	174.96	34.95	30.11		5.64		4.86
Sao Tome and Principe	2.46	26.25	17.38		1.87		1.24
Senegal	165.98	45.16	30.94		4.38		3.00
Sierra Leone	184.08	17.28	12.65		2.72		1.99
Solomon Islands	4.47	45.92	25.81		3.02		1.70
Somalia	476.59	14.98	10.60		0.20		0.14
South Africa	320.65	156.96	60.73		9.93		3.84
Sudan	772.94	43.67	22.32		1.43		0.73
Swaziland	15.91	36.47	20.27		4.18		2.32
Tajikistan	107.30	51.12	39.35		8.40		6.46
Tanzania, United Republic of	634.76	25.09	18.85		4.37		3.28
Togo	117.10	30.21	22.99		0.67		0.51
Turkmenistan	46.83	91.93	42.53		1.41		0.65
Uganda	654.10	34.33	27.95		6.28		5.11
Uzbekistan	241.88	67.19	46.57		3.91		2.71
Viet Nam	214.59	85.53	35.54		5.36		2.23
Yemen	303.25	74.12	44.68		4.96		2.99
Zambia	311.04	26.27	19.94		1.79		1.36
Zimbabwe	238.01	11.37	6.71		1.44		0.85
Total	39 613.61						
Median (IQR)	212.37 (82.00–493.51)	38.45 (26.25–87.68)	26.67 (17.75–46.43)				

DALYs – disability adjusted life years, GDP – gross domestic product
