# Supplementary material for: Cost–effectiveness analysis of revised WHO guidelines for management of childhood pneumonia in 74 Countdown countries
Source: J Glob Health. 2017 Mar 7;7(1):010409. doi: 10.7189/jogh.07.010409 (PMC5344007; doi:10.7189/jogh.07.010409)
Supplement: Online Supplementary Document [file jogh-07-010409-s001.pdf]

Online Supplementary Document

Zhang et al. Cost–effectiveness analysis of revised WHO guidelines for management of childhood pneumonia in 74 Countdown countries

J Glob Health 2017;7:010409

Supplementary material for "Cost-effectiveness analysis of 2013 WHO guidelines for management of childhood pneumonia in 74 countdown countries"

Supplementary 1 table: Total cost savings at national level by HIV prevalence and CCM implementation status 3
Supplementary 2 table: Total cost of pneumonia treatment in a high cost scenario 5
Supplementary 3 table: Total cost of pneumonia treatment at national level by HIV status in a high cost scenario 6
Supplementary 4 table: Total DALYs averted and cost-effectiveness of implementing 2013 guidelines in a high cost scenario 9
Supplementary 5 table: Total cost of pneumonia treatment in a low cost scenario 11
Supplementary 6 table: Total cost of pneumonia treatment by HIV status in a low cost scenario 12
Supplementary 7 table: Total DALYs averted and cost-effectiveness of implementing 2013 guidelines in a low cost scenario 15
Supplementary 8 table: Total cost of pneumonia treatment in 2013 using effective access to care scenario 17
Supplementary 9 table: Total cost of pneumonia treatment by HIV status using effective access to care scenario 18
Supplementary 10 table: Total DALYs averted and cost-effectiveness of implementing 2013 guidelines using effective access to care scenario 21
Supplementary 11 table: Total cost of pneumonia treatment in 2013 using unpublished data on proportion of clinical signs in children with pneumonia in a high HIV burden setting 23
Supplementary 12 table: Total cost of pneumonia treatment by HIV status using unpublished data on proportion of clinical signs in children with pneumonia in high HIV burden settings 24
Supplementary 13 table: Total DALYs averted and cost-effectiveness of implementing 2013 guidelines using unpublished data on proportion of clinical signs in children with pneumonia in high HIV burden settings 27
Supplementary 14 table: Total cost of pneumonia treatment in a high cost less effective scenario 29
Supplementary 15 table: Total cost of pneumonia treatment at national level by HIV status in a high cost less effective scenario 30
Supplementary 16 table: Total DALYs averted and cost-effectiveness of implementing 2013 guidelines in a high cost less effective scenario 33
Supplementary 17 table: Total DALYs averted and cost-effectiveness of implementing 2013 guidelines in a less effective scenario 35
Supplementary 18 table: Total DALYs averted and cost-effectiveness of implementing 2013 guidelines in not cost-effective scenario 37
Supplementary 19 table: Years of Life Saved and deaths averted in each country by implementing 2013 guidelines 39

Supplementary 1 table: Total cost savings at national level by HIV prevalence and CCM implementation status

HIV Prevalence	CCM implementation	Country	Total Cost Savings = 2013 Guidelines - 2005 Guidelines (Thousands, US$ 2013)		
			HIV+	HIV-	Total
Low HIV Prevalence	Yes	Afghanistan	0.18	-7,545.94	-7,545.77
		Azerbaijan	14.00	-4,749.49	-4,735.49
		Bangladesh	0.27	-21,719.94	-21,719.66
		China	32.55	-199,454.33	-199,421.77
		Eritrea	4.39	-1,587.07	-1,582.68
		Guatemala	0.81	-1,473.90	-1,473.09
		India	523.54	-470,813.10	-470,289.56
		Indonesia	23.96	-28,014.19	-27,990.23
		Korea, Democratic People's Republic	0.07	-3,873.31	-3,873.24
		Kyrgyzstan	0.06	-881.16	-881.09
		Lao People's Democratic Republic	1.56	-1,519.98	-1,518.42
		Madagascar	2.24	-3,441.29	-3,439.05
		Mexico	19.81	-61,334.08	-61,314.27
		Myanmar	9.03	-5,822.95	-5,813.92
		Nepal	0.99	-2,368.22	-2,367.23
		Niger	1.78	-3,419.43	-3,417.64
		Pakistan	2.98	-34,518.97	-34,516.00
		Papua New Guinea	4.47	-1,542.58	-1,538.11
		Peru	7.75	-7,412.32	-7,404.57
		Philippines	0.92	-27,310.63	-27,309.71
		Senegal	5.08	-2,365.19	-2,360.11
		Somalia	0.08	-2,087.11	-2,087.03
		Tajikistan	0.56	-1,263.21	-1,262.65
		Turkmenistan	1.50	-2,314.97	-2,313.48
		Uzbekistan	5.27	-4,994.20	-4,988.92
		Yemen	2.66	-8,927.94	-8,925.28
		Total	666.52	-910,755.50	-910,088.98
	No	Bolivia (Plurinational State of)	1.04	-1,370.51	-1,369.47
		Brazil	4.36	-21,311.35	-21,306.99
		Cambodia	2.73	-1,343.13	-1,340.40
		Comoros	0.01	-174.55	-174.54
		Egypt	0.30	-10,726.93	-10,726.63
		Iraq	77.51	-16,318.11	-16,240.60
		Morocco	1.29	-5,309.51	-5,308.22
		Solomon Islands	0.12	-101.91	-101.79
		Viet Nam	11.98	-11,371.87	-11,359.88
		Total	99.34	-68,027.86	-67,928.52
High HIV Prevalence	Yes	Benin	4.50	-1,602.96	-1,598.46
		Burkina Faso	8.07	-4,504.05	-4,495.98
		Burundi	0.38	-695.93	-695.55
		Cameroon	60.82	-4,271.57	-4,210.75
		Congo	45.18	-2,197.63	-2,152.46
		Congo, Democratic Republic	-1.38	-9,019.26	-9,020.64
		Ethiopia	15.30	-8,146.25	-8,130.95
		Gambia	0.99	-293.42	-292.43
		Ghana	21.87	-3,403.20	-3,381.33
		Guinea	4.58	-1,721.13	-1,716.55
		Liberia	0.13	-483.09	-482.96
		Malawi	12.89	-1,102.01	-1,089.12
		Mali	6.83	-3,056.12	-3,049.30
		Mauritania	2.22	-842.57	-840.35
		Mozambique	67.37	-3,174.15	-3,106.78
		Nigeria	641.43	-40,432.83	-39,791.39
		Rwanda	3.71	-849.31	-845.59

HIV Prevalence	CCM implementation	Country	Total Cost Savings = 2013 Guidelines - 2005 Guidelines (Thousands, US$ 2013)		
			HIV+	HIV-	Total
		Sierra Leone	1.01	-854.28	-853.27
		Togo	5.67	-851.70	-846.03
		Uganda	28.17	-4,199.12	-4,170.95
		Zambia	106.51	-2,073.81	-1,967.29
		Total	1,036.26	-93,774.39	-92,738.14
	No	Angola	219.62	-18,035.08	-17,815.46
		Botswana	118.48	-1,723.48	-1,605.01
		Central African Republic	5.71	152.92	158.64
		Chad	28.48	-3,367.77	-3,339.29
		Côte d'Ivoire	72.20	-5,733.55	-5,661.34
		Djibouti	1.65	-203.13	-201.48
		Equatorial Guinea	261.41	-3,605.83	-3,344.42
		Gabon	58.04	-2,104.47	-2,046.44
		Guinea-Bissau	2.53	-270.79	-268.26
		Haiti	3.97	-953.21	-949.24
		Kenya	81.52	-5,064.06	-4,982.54
		Lesotho	22.78	-252.58	-229.79
		Sao Tome and Principe	0.20	-21.89	-21.69
		South Africa	3,361.71	-32,904.18	-29,542.47
		Sudan	15.84	-16,515.01	-16,499.17
		Swaziland	50.55	-287.93	-237.37
		Tanzania, United Republic of	44.03	-3,978.01	-3,933.98
		Zimbabwe	50.07	-1,130.81	-1,080.74
		Total	4,398.79	-95,998.86	-91,600.07
		**Subgroup (No. of countries)**	**HIV +**	**HIV-**	**Total Cost Saving**
		**CCM (47)**	**1,702.77**	**-1,004,529.89**	**-1,002,827.12**
		**NO-CCM (27)**	**4,498.13**	**-164,026.72**	**-159,528.59**
		**Low HIV Prevalence (34)**	**765.86**	**-978,783.36**	**-978,017.50**
		**High HIV Prevalence (40)**	**5,435.05**	**-189,773.25**	**-184,338.20**
		**TOTAL**	**6,200.91**	**-1,168,556.61**	**-1,162,355.70**

I. High cost scenario

High cost scenario assumptions:

1. CCM Coverage: 100% coverage of rural population in countries with CCM
2. Unit cost of medicine changed based on review by Zhang et al 2015 (in press)

All other assumptions remain the same as the main model.

Supplementary 2 table: Total cost of pneumonia treatment in a high cost scenario

Delivery Levels	Total Cost for Pneumonia Treatment in 2013 (Billions, in US$ 2013)				
	2005 Guidelines		2013 Guidelines(		2013/2005%
	Total Cost	%Total	Total Cost	%Total	
**Community**	2.52	59.5%	2.51	83.3%	99.4%
**First Level Facility**	0.15	3.5%	0.17	5.8%	117.2%
**Hospital**	1.57	37.0%	0.33	10.9%	21.0%
**Total cost**	4.24	100%	3.01	100.0%	71.0%

Supplementary 3 table: Total cost of pneumonia treatment at national level by HIV status in a high cost scenario

Country	2005 Guidelines (Thousands, US$)			2013 Guidelines(Thousands, US$)			Total Cost Savings (Thousands, US$)	2013 Guidelines		National healthcare expenditure per capita (US$)
	HIV+	HIV-	Total	HIV+	HIV-	Total		Total cost per capita (US$)	Proportion of national healthcare expenditure per capita	
Afghanistan	4.79	41,353.93	41,358.72	4.98	32,271.29	32,276.27	9,082.44	1.06	1.89	55.93
Azerbaijan	39.61	11,549.23	11,588.85	53.66	6,693.26	6,746.92	4,841.93	0.72	0.20	356.92
Bangladesh	3.67	141,956.16	141,959.83	3.96	117,337.27	117,341.22	24,618.61	0.75	2.82	26.54
China	89.67	925,232.53	925,322.21	122.36	720,361.46	720,483.83	204,838.38	0.52	0.19	278.02
Eritrea	19.43	7,012.79	7,032.22	23.88	5,325.57	5,349.45	1,682.77	0.84	6.08	13.90
Guatemala	20.64	9,894.60	9,915.24	21.54	8,188.10	8,209.65	1,705.59	0.53	0.25	213.94
India	1,853.51	1,466,513.18	1,468,366.70	2,381.22	973,976.43	976,357.66	492,009.04	0.78	1.32	59.10
Indonesia	143.61	162,156.09	162,299.70	168.01	131,798.36	131,966.38	30,333.32	0.53	0.56	94.99
Korea, Democratic People's Republic	0.23	14,801.09	14,801.32	0.30	10,760.73	10,761.03	4,040.29	0.43	1.15	37.62
Kyrgyzstan	0.50	4,783.63	4,784.14	0.57	3,809.90	3,810.47	973.66	0.69	0.96	71.25
Lao People's Democratic Republic	10.51	6,499.48	6,509.99	12.11	4,838.86	4,850.96	1,659.03	0.72	1.95	36.74
Madagascar	65.36	20,781.75	20,847.11	67.89	16,767.74	16,835.62	4,011.49	0.73	3.87	18.98
Mexico	45.88	105,690.21	105,736.10	65.74	43,507.76	43,573.50	62,162.60	0.36	0.06	619.62
Myanmar	79.71	43,523.94	43,603.64	89.04	37,059.04	37,148.07	6,455.57	0.70	3.09	22.54
Nepal	17.91	26,244.24	26,262.15	18.97	23,515.89	23,534.86	2,727.29	0.85	2.57	33.00
Niger	88.48	20,833.71	20,922.19	90.66	16,739.41	16,830.06	4,092.12	0.94	4.69	20.11
Pakistan	23.92	174,406.13	174,430.05	26.98	136,168.76	136,195.73	38,234.32	0.75	2.52	29.70
Papua New Guinea	23.51	8,413.58	8,437.08	28.05	6,752.68	6,780.73	1,656.35	0.93	1.17	78.87
Peru	20.40	16,362.31	16,382.71	28.18	8,773.92	8,802.10	7,580.61	0.29	0.10	288.95
Philippines	4.03	88,615.51	88,619.54	4.96	59,653.38	59,658.34	28,961.20	0.61	0.63	96.51
Senegal	43.30	11,593.75	11,637.05	48.54	8,972.44	9,020.97	2,616.08	0.64	0.95	66.97
Somalia	56.99	10,422.14	10,479.13	57.33	7,903.28	7,960.61	2,518.52	0.76	19.65	3.86
Tajikistan	5.71	8,204.51	8,210.22	6.29	6,779.03	6,785.32	1,424.90	0.83	1.53	54.08

Country	2005 Guidelines (Thousands, US$)			2013 Guidelines(Thousands, US$)			Total Cost Savings (Thousands, US$)	2013 Guidelines		National healthcare expenditure per capita (US$)
	HIV+	HIV-	Total	HIV+	HIV-	Total		Total cost per capita (US$)	Proportion of national healthcare expenditure per capita	
**Turkmenistan**	4.31	5,680.81	5,685.12	5.81	3,296.20	3,302.01	2,383.11	0.63	0.49	129.07
**Uzbekistan**	29.98	25,395.01	25,424.99	35.34	19,996.99	20,032.33	5,392.65	0.69	0.78	88.40
**Yemen**	16.06	30,290.64	30,306.70	18.77	20,594.14	20,612.91	9,693.79	0.84	0.96	88.35
**Bolivia (Plurinational State of)**	4.59	2,063.32	2,067.90	5.64	615.55	621.19	1,446.71	0.06	0.05	118.12
**Brazil**	16.95	30,751.76	30,768.72	21.35	8,372.61	8,393.97	22,374.75	0.04	0.004	1,120.56
**Cambodia**	29.91	2,325.99	2,355.90	32.75	829.63	862.38	1,493.52	0.06	0.12	51.21
**Comoros**	0.28	695.00	695.27	0.29	498.13	498.42	196.86	0.77	1.81	42.55
**Egypt**	1.18	15,579.34	15,580.52	1.48	4,329.63	4,331.11	11,249.40	0.06	0.04	136.58
**Iraq**	334.43	31,006.42	31,340.85	412.44	14,056.13	14,468.57	16,872.28	0.47	0.14	331.59
**Morocco**	6.77	11,527.88	11,534.65	8.07	5,969.74	5,977.81	5,556.84	0.20	0.11	185.89
**Solomon Islands**	0.94	217.12	218.06	1.06	106.71	107.77	110.29	0.21	0.16	133.99
**Viet Nam**	86.43	19,745.62	19,832.05	98.67	7,390.32	7,488.99	12,343.06	0.09	0.09	94.81
**Benin**	53.98	7,896.64	7,950.62	58.70	6,091.59	6,150.29	1,800.33	0.60	1.62	36.70
**Burkina Faso**	158.95	19,296.58	19,455.54	167.71	14,107.88	14,275.59	5,179.95	0.84	2.27	37.21
**Burundi**	79.42	10,241.17	10,320.58	80.17	9,384.00	9,464.17	856.42	0.93	3.99	23.37
**Cameroon**	424.31	16,442.50	16,866.81	486.61	11,768.36	12,254.97	4,611.84	0.55	0.81	68.24
**Congo**	155.34	4,570.15	4,725.49	200.84	2,278.13	2,478.97	2,246.52	0.56	0.64	87.38
**Congo, Democratic Republic**	539.44	58,209.59	58,749.03	540.63	47,401.89	47,942.52	10,806.51	0.71	3.60	19.73
**Ethiopia**	692.27	88,359.51	89,051.78	710.79	78,813.55	79,524.34	9,527.45	0.85	5.09	16.61
**Gambia**	15.75	1,270.27	1,286.02	16.80	935.08	951.88	334.14	0.51	1.88	27.41
**Ghana**	138.53	17,060.18	17,198.70	160.85	13,358.45	13,519.30	3,679.40	0.52	0.70	75.02
**Guinea**	116.96	10,434.55	10,551.51	122.03	8,428.03	8,550.06	2,001.45	0.73	2.45	29.73
**Liberia**	21.45	4,081.57	4,103.02	21.67	3,501.68	3,523.35	579.67	0.57	1.03	54.90
**Malawi**	608.95	15,428.66	16,037.61	624.58	14,114.16	14,738.74	1,298.87	0.90	2.91	30.93
**Mali**	122.94	15,410.62	15,533.56	130.26	11,877.86	12,008.11	3,525.45	0.78	1.76	44.57
**Mauritania**	20.47	3,798.48	3,818.95	22.76	2,857.39	2,880.15	938.80	0.74	1.28	57.71

Country	2005 Guidelines (Thousands, US$)			2013 Guidelines(Thousands, US$)			Total Cost Savings (Thousands, US$)	2013 Guidelines		National healthcare expenditure per capita (US$)
	HIV+	HIV-	Total	HIV+	HIV-	Total		Total cost per capita (US$)	Proportion of national healthcare expenditure per capita	
**Mozambique**	1,795.55	25,845.05	27,640.59	1,869.72	22,021.97	23,891.69	3,748.91	0.92	2.63	35.22
**Nigeria**	4,545.67	163,610.14	168,155.80	5,200.30	119,295.14	124,495.44	43,660.37	0.72	0.90	79.56
**Rwanda**	71.63	10,673.84	10,745.47	75.64	9,691.49	9,767.13	978.34	0.83	1.32	62.72
**Sierra Leone**	50.23	5,062.17	5,112.40	51.46	4,055.25	4,106.71	1,005.69	0.67	0.98	68.54
**Togo**	125.19	5,566.03	5,691.22	131.40	4,579.61	4,711.01	980.21	0.69	1.54	44.88
**Uganda**	950.05	37,474.71	38,424.76	972.01	32,586.46	33,558.48	4,866.29	0.89	2.11	42.40
**Zambia**	786.80	11,860.99	12,647.79	891.27	9,584.90	10,476.17	2,171.62	0.72	0.83	87.22
**Angola**	635.97	24,583.14	25,219.12	856.57	6,024.95	6,881.52	18,337.60	0.32	0.17	186.26
**Botswana**	283.79	2,246.15	2,529.95	402.54	494.63	897.17	1,632.77	0.42	0.10	431.91
**Central African Republic**	574.13	7,560.54	8,134.67	580.58	7,584.76	8,165.34	-30.66	1.77	9.67	18.28
**Chad**	355.45	5,570.50	5,925.95	385.29	1,774.13	2,159.42	3,766.53	0.17	0.48	35.20
**Côte d'Ivoire**	669.09	10,846.73	11,515.81	743.44	4,487.67	5,231.11	6,284.71	0.26	0.32	79.43
**Djibouti**	11.96	325.79	337.75	13.64	103.45	117.09	220.66	0.13	0.13	105.20
**Equatorial Guinea**	521.30	4,561.92	5,083.22	782.84	942.26	1,725.10	3,358.11	2.15	0.17	1,236.15
**Gabon**	132.09	2,732.22	2,864.31	190.23	601.46	791.69	2,072.62	0.46	0.13	358.30
**Guinea-Bissau**	50.33	464.76	515.09	53.06	153.73	206.79	308.29	0.12	0.32	37.18
**Haiti**	66.57	1,965.85	2,032.42	70.79	875.70	946.49	1,085.94	0.09	0.16	57.66
**Kenya**	1,251.93	13,210.48	14,462.41	1,337.33	7,428.01	8,765.34	5,697.07	0.20	0.54	36.25
**Lesotho**	203.37	395.33	598.70	226.89	115.39	342.27	256.43	0.16	0.11	141.10
**Sao Tome and Principe**	1.89	66.22	68.12	2.10	42.04	44.14	23.98	0.23	0.19	117.44
**South Africa**	8,288.46	43,342.47	51,630.93	11,658.89	9,847.35	21,506.24	30,124.69	0.38	0.06	689.27
**Sudan**	146.87	36,283.41	36,430.28	163.14	17,989.64	18,152.78	18,277.51	0.48	0.46	103.53
**Swaziland**	187.34	430.54	617.88	238.27	129.71	367.98	249.90	0.28	0.11	264.81
**Tanzania, United Republic of**	1,445.94	15,843.48	17,289.42	1,494.17	11,086.63	12,580.80	4,708.63	0.25	0.68	37.33
**Zimbabwe**	864.96	2,239.95	3,104.91	918.25	945.63	1,863.88	1,241.04	0.13	0.34	38.38
**Total**	30,358.51	4,207,386.31	4,237,744.82	36,621.06	2,971,370.34	3,007,991.41	1,229,753.42			

Supplementary 4 table: Total DALYs averted and cost-effectiveness of implementing 2013 guidelines in a high cost scenario

Country	Total DALY averted (Thousands)	Cost per DALY averted (US$,2013)		Percentage of cost per DALY averted of GDP per capita (%)	
		2005	2013	2005	2013
**Afghanistan**	875.44	47.24	36.87	7.62	5.95
**Azerbaijan**	45.64	253.91	147.82	3.43	2.00
**Bangladesh**	910.09	155.98	128.93	20.87	17.25
**China**	1,752.85	527.90	411.04	8.53	6.64
**Eritrea**	98.98	71.05	54.04	14.09	10.72
**Guatemala**	132.69	74.73	61.87	2.23	1.85
**India**	8,522.64	172.29	114.56	11.57	7.69
**Indonesia**	1,137.93	142.63	115.97	4.01	3.26
**Korea, Democratic People's Republic**	82.09	180.31	131.09	35.63	25.91
**Kyrgyzstan**	24.97	191.60	152.61	16.52	13.16
**Lao People's Democratic Republic**	124.61	52.24	38.93	3.73	2.78
**Madagascar**	370.93	56.20	45.39	12.56	10.14
**Mexico**	212.37	497.88	205.17	5.11	2.10
**Myanmar**	327.07	133.32	113.58	11.65	9.93
**Nepal**	165.78	158.41	141.96	22.42	20.09
**Niger**	635.69	32.91	26.48	8.60	6.92
**Pakistan**	3,212.46	54.30	42.40	4.21	3.29
**Papua New Guinea**	96.55	87.39	70.23	4.00	3.22
**Peru**	57.94	282.76	151.92	4.31	2.31
**Philippines**	595.98	148.70	100.10	5.75	3.87
**Senegal**	165.98	70.11	54.35	6.80	5.27
**Somalia**	476.59	21.99	16.70	0.29	0.22
**Tajikistan**	107.30	76.52	63.24	12.57	10.39
**Turkmenistan**	46.83	121.40	70.51	1.86	1.08
**Uzbekistan**	241.88	105.11	82.82	6.12	4.82
**Yemen**	303.25	99.94	67.97	6.69	4.55
**Bolivia (Plurinational State of)**	81.97	25.23	7.97	0.98	0.31
**Brazil**	167.92	183.23	50.14	1.62	0.44
**Cambodia**	120.25	19.59	7.72	2.07	0.82
**Comoros**	13.03	53.36	43.49	6.43	5.24
**Egypt**	227.57	68.47	19.98	2.15	0.63
**Iraq**	265.05	118.25	60.16	1.83	0.93
**Morocco**	151.78	75.99	43.94	2.62	1.51
**Solomon Islands**	4.47	48.78	26.77	3.21	1.76
**Viet Nam**	214.59	92.42	37.85	5.79	2.37
**Benin**	191.87	41.44	32.05	5.51	4.26
**Burkina Faso**	390.63	49.81	36.55	7.85	5.76
**Burundi**	250.15	41.26	37.83	16.44	15.08
**Cameroon**	499.15	33.79	24.55	2.93	2.13
**Congo**	37.44	126.21	66.21	4.00	2.10
**Congo,**	1,770.31	33.19	27.08	12.20	9.96

Country	Total DALY averted (Thousands)	Cost per DALY averted (US$,2013)		Percentage of cost per DALY averted of GDP per capita (%)	
		2005	2013	2005	2013
**Democratic Republic Ethiopia**	1,568.40	56.78	50.70	12.07	10.78
**Gambia**	29.69	43.31	32.06	8.46	6.26
**Ghana**	352.92	48.73	38.31	3.04	2.39
**Guinea**	224.05	47.09	38.16	7.97	6.46
**Liberia**	64.60	63.52	54.54	15.06	12.93
**Malawi**	215.99	74.25	68.24	27.70	25.46
**Mali**	515.92	30.11	23.28	4.34	3.35
**Mauritania**	80.87	47.22	35.61	4.27	3.22
**Mozambique**	423.41	65.28	56.43	11.28	9.75
**Nigeria**	4,499.06	37.38	27.67	2.40	1.78
**Rwanda**	174.96	61.42	55.82	9.91	9.00
**Sierra Leone**	184.08	27.77	22.31	4.37	3.51
**Togo**	117.10	48.60	40.23	1.08	0.90
**Uganda**	654.10	58.74	51.30	10.74	9.38
**Zambia**	311.04	40.66	33.68	2.77	2.29
**Angola**	971.25	25.97	7.00	0.47	0.13
**Botswana**	9.75	259.48	87.27	3.61	1.21
**Central African Republic**	113.76	71.51	71.74	15.13	15.18
**Chad**	516.30	11.48	4.15	1.30	0.47
**Côte d'Ivoire**	391.80	29.39	13.27	2.36	1.07
**Djibouti**	11.01	30.67	10.57	2.89	1.00
**Equatorial Guinea**	14.30	355.51	113.60	1.48	0.47
**Gabon**	16.33	175.43	47.11	1.53	0.41
**Guinea-Bissau**	41.79	12.32	4.91	2.28	0.91
**Haiti**	172.73	11.77	5.47	1.53	0.71
**Kenya**	817.99	17.68	10.67	2.04	1.23
**Lesotho**	24.95	23.99	13.29	2.01	1.11
**Sao Tome and Principe**	2.46	27.72	17.93	1.98	1.28
**South Africa**	320.65	161.02	62.97	10.19	3.99
**Sudan**	772.94	47.13	23.48	1.55	0.77
**Swaziland**	15.91	38.83	21.84	4.45	2.50
**Tanzania, United Republic of**	634.76	27.24	19.78	4.74	3.44
**Zimbabwe**	238.01	13.05	7.72	1.66	0.98
**Total**	39,613.61				
**Median (IQR)**	212.37 (82.00-493.51)	56.20 (34.69-120.61)			

II. Low cost scenario

Low cost scenario Assumptions:

1. CCM Coverage: coverage reduced to 50% coverage of rural population in countries with CCM, the remaining 50% were treated in health centre.
2. 1/5000 ratio of Community Health Works/ rural population\

All other assumptions are the same as the main model.

Supplementary 5 table: Total cost of pneumonia treatment in a low cost scenario

Delivery Levels	Total Cost for Pneumonia Treatments in 2013 (Billions, in US$ 2013)				
	2005 Guidelines		2013 Guidelines		2013/2005 %
	Total Cost	%Total	Total Cost	%Total	
**Community**	0.25	12.9%	0.25	32.6%	101.4%
**First Level Facility**	0.18	9.3%	0.23	29.0%	126.0%
**Hospital**	1.51	77.8%	0.30	38.4%	19.9%
**Total cost**	1.94	100%	0.78	100.0%	40.3%

Supplementary 6 table: Total cost of pneumonia treatment by HIV status in a low cost scenario

Country	2005 Guidelines (Thousands)			2013 Guidelines(Thousands)			Total Cost Savings (Thousands)	2013 Guidelines	
	HIV+	HIV-	Total	HIV+	HIV-	Total		Total cost per capita	Proportion of national healthcare expenditure per capita
**Afghanistan**	4.78	34,135.63	34,140.41	4.95	26,589.69	26,594.64	7,545.77	0.57	1.03
**Azerbaijan**	39.30	9,656.73	9,696.03	53.31	4,907.24	4,960.54	4,735.49	0.35	0.10
**Bangladesh**	3.03	84,736.32	84,739.35	3.30	63,016.38	63,019.68	21,719.66	0.12	0.47
**China**	83.52	586,413.86	586,497.37	116.07	386,959.53	387,075.60	199,421.77	0.09	0.03
**Eritrea**	17.15	4,497.40	4,514.55	21.54	2,910.33	2,931.87	1,582.68	0.16	1.13
**Guatemala**	16.60	5,851.12	5,867.72	17.41	4,377.21	4,394.63	1,473.09	0.09	0.04
**India**	1,669.57	1,024,440.35	1,026,109.93	2,193.12	553,627.25	555,820.37	470,289.56	0.18	0.30
**Indonesia**	125.39	100,469.43	100,594.82	149.35	72,455.25	72,604.59	27,990.23	0.10	0.10
**Korea, Democratic People's Republic**	0.21	9,792.33	9,792.54	0.27	5,919.03	5,919.30	3,873.24	0.08	0.22
**Kyrgyzstan**	0.43	2,949.80	2,950.22	0.49	2,068.64	2,069.13	881.09	0.12	0.17
**Lao People's Democratic Republic**	8.92	4,182.93	4,191.84	10.47	2,662.95	2,673.42	1,518.42	0.14	0.38
**Madagascar**	54.14	12,884.32	12,938.46	56.38	9,443.03	9,499.41	3,439.05	0.15	0.80
**Mexico**	44.11	91,613.44	91,657.55	63.93	30,279.36	30,343.29	61,314.27	0.16	0.03
**Myanmar**	67.27	25,527.24	25,594.51	76.30	19,704.29	19,780.59	5,813.92	0.11	0.49
**Nepal**	15.35	14,976.83	14,992.18	16.34	12,608.62	12,624.96	2,367.23	0.13	0.40
**Niger**	80.04	14,302.62	14,382.67	81.83	10,883.19	10,965.02	3,417.64	0.30	1.47
**Pakistan**	21.97	120,173.52	120,195.50	24.95	85,654.55	85,679.50	34,516.00	0.22	0.75
**Papua New Guinea**	20.99	5,268.41	5,289.40	25.47	3,725.83	3,751.30	1,538.11	0.17	0.22
**Peru**	19.10	12,763.31	12,782.41	26.85	5,350.99	5,377.84	7,404.57	0.09	0.03
**Philippines**	3.61	62,967.52	62,971.13	4.54	35,656.88	35,661.42	27,309.71	0.16	0.17
**Senegal**	37.10	7,458.85	7,495.95	42.18	5,093.66	5,135.84	2,360.11	0.14	0.21
**Somalia**	47.85	7,093.02	7,140.86	47.93	5,005.91	5,053.84	2,087.03	0.24	6.22
**Tajikistan**	5.36	5,479.34	5,484.70	5.92	4,216.14	4,222.05	1,262.65	0.23	0.42
**Turkmenistan**	4.00	4,301.07	4,305.08	5.50	1,986.10	1,991.60	2,313.48	0.18	0.14

Country	2005 Guidelines (Thousands)			2013 Guidelines(Thousands)			Total Cost Savings (Thousands)	2013 Guidelines	
	HIV+	HIV-	Total	HIV+	HIV-	Total		Total cost per capita	Proportion of national healthcare expenditure per capita
**Uzbekistan**	26.54	16,226.31	16,252.84	31.81	11,232.11	11,263.92	4,988.92	0.14	0.16
**Yemen**	14.65	22,461.41	22,476.07	17.31	13,533.47	13,550.78	8,925.28	0.29	0.33
**Bolivia (Plurinational State of)**	4.06	1,948.07	1,952.13	5.10	577.56	582.66	1,369.47	0.06	0.05
**Brazil**	15.10	29,158.90	29,174.00	19.46	7,847.54	7,867.01	21,306.99	0.04	0.004
**Cambodia**	24.87	2,097.41	2,122.28	27.60	754.28	781.88	1,340.40	0.06	0.11
**Comoros**	0.25	661.70	661.95	0.26	487.15	487.41	174.54	0.76	1.78
**Egypt**	1.05	14,799.49	14,800.54	1.35	4,072.57	4,073.92	10,726.63	0.05	0.04
**Iraq**	310.48	30,063.38	30,373.86	387.99	13,745.27	14,133.26	16,240.60	0.46	0.14
**Morocco**	6.27	11,156.99	11,163.26	7.55	5,847.48	5,855.04	5,308.22	0.20	0.11
**Solomon Islands**	0.83	204.44	205.27	0.95	102.53	103.48	101.79	0.21	0.15
**Viet Nam**	74.25	18,278.60	18,352.85	86.23	6,906.74	6,992.97	11,359.88	0.08	0.09
**Benin**	43.96	4,878.91	4,922.87	48.47	3,275.94	3,324.41	1,598.46	0.11	0.30
**Burkina Faso**	132.04	12,763.14	12,895.19	140.12	8,259.09	8,399.21	4,495.98	0.21	0.57
**Burundi**	75.95	6,156.61	6,232.56	76.33	5,460.68	5,537.02	695.55	0.20	0.85
**Cameroon**	355.52	10,737.84	11,093.36	416.34	6,466.27	6,882.61	4,210.75	0.12	0.18
**Congo**	140.61	3,670.67	3,811.28	185.79	1,473.04	1,658.82	2,152.46	0.23	0.27
**Congo, Democratic Republic**	429.87	34,908.51	35,338.38	428.49	25,889.25	26,317.74	9,020.64	0.13	0.68
**Ethiopia**	552.14	48,956.23	49,508.37	567.44	40,809.98	41,377.42	8,130.95	0.12	0.71
**Gambia**	13.10	846.88	859.98	14.09	553.47	567.55	292.43	0.14	0.51
**Ghana**	118.24	10,671.04	10,789.28	140.10	7,267.84	7,407.95	3,381.33	0.10	0.13
**Guinea**	99.32	6,646.82	6,746.14	103.90	4,925.69	5,029.59	1,716.55	0.18	0.60
**Liberia**	17.86	2,465.72	2,483.58	17.99	1,982.63	2,000.62	482.96	0.12	0.22
**Malawi**	543.94	8,873.99	9,417.94	556.83	7,771.98	8,328.82	1,089.12	0.18	0.59
**Mali**	108.31	10,637.06	10,745.37	115.13	7,580.94	7,696.07	3,049.30	0.25	0.56
**Mauritania**	18.69	2,740.12	2,758.81	20.91	1,897.56	1,918.47	840.35	0.27	0.46
**Mozambique**	1,774.32	18,813.27	20,587.58	1,841.68	15,639.12	17,480.80	3,106.78	0.41	1.16

Country	2005 Guidelines (Thousands)			2013 Guidelines(Thousands)			Total Cost Savings (Thousands)	2013 Guidelines	
	HIV+	HIV-	Total	HIV+	HIV-	Total		Total cost per capita	Proportion of national healthcare expenditure per capita
**Nigeria**	4,233.83	123,748.56	127,982.39	4,875.27	83,315.73	88,190.99	39,791.39	0.31	0.39
**Rwanda**	62.14	6,052.42	6,114.56	65.85	5,203.11	5,268.96	845.59	0.13	0.21
**Sierra Leone**	41.06	3,140.00	3,181.06	42.06	2,285.72	2,327.79	853.27	0.15	0.21
**Togo**	105.00	3,433.18	3,538.18	110.67	2,581.48	2,692.15	846.03	0.15	0.34
**Uganda**	788.10	21,666.61	22,454.70	816.27	17,467.48	18,283.75	4,170.95	0.16	0.37
**Zambia**	690.95	7,479.67	8,170.62	797.46	5,405.86	6,203.32	1,967.29	0.19	0.22
**Angola**	588.79	23,802.80	24,391.59	808.42	5,767.72	6,576.13	17,815.46	0.30	0.16
**Botswana**	270.49	2,204.32	2,474.81	388.96	480.84	869.80	1,605.01	0.41	0.09
**Central African Republic**	538.84	7,368.55	7,907.39	544.55	7,521.47	8,066.02	-158.64	1.75	9.55
**Chad**	289.80	4,931.14	5,220.94	318.28	1,563.37	1,881.65	3,339.29	0.15	0.41
**Côte d'Ivoire**	565.27	9,913.63	10,478.90	637.47	4,180.08	4,817.55	5,661.34	0.24	0.30
**Djibouti**	10.07	297.13	307.20	11.72	94.00	105.72	201.48	0.12	0.11
**Equatorial Guinea**	514.83	4,541.29	5,056.12	776.24	935.46	1,711.70	3,344.42	2.13	0.17
**Gabon**	127.20	2,693.01	2,820.21	185.24	588.53	773.77	2,046.44	0.45	0.13
**Guinea-Bissau**	40.40	404.74	445.14	42.93	133.95	176.88	268.26	0.10	0.28
**Haiti**	54.97	1,761.58	1,816.55	58.94	808.36	867.31	949.24	0.08	0.15
**Kenya**	1,064.89	12,138.81	13,203.70	1,146.41	7,074.75	8,221.16	4,982.54	0.18	0.51
**Lesotho**	168.05	354.51	522.56	190.83	101.93	292.76	229.79	0.14	0.10
**Sao Tome and Principe**	1.68	62.80	64.49	1.89	40.91	42.80	21.69	0.22	0.19
**South Africa**	7,868.39	42,460.96	50,329.35	11,230.10	9,556.77	20,786.88	29,542.47	0.37	0.05
**Sudan**	126.39	33,629.99	33,756.38	142.23	17,114.97	17,257.20	16,499.17	0.45	0.44
**Swaziland**	169.06	411.29	580.35	219.61	123.37	342.98	237.37	0.26	0.10
**Tanzania, United Republic of**	1,243.91	14,681.66	15,925.57	1,287.95	10,703.65	11,991.59	3,933.98	0.24	0.65
**Zimbabwe**	709.98	1,996.04	2,706.02	760.05	865.22	1,625.27	1,080.74	0.11	0.29
**Total**	27,566.10	2,911,933.58	2,939,499.68	33,767.00	1,743,376.97	1,777,143.97	1,162,355.70		

Supplementary 7 table: Total DALYs averted and cost-effectiveness of implementing 2013 guidelines in a low cost scenario

Country	Total DALY averted (Thousands)	Cost per DALY averted (US$,2013)		Percentage of cost per DALY averted in GDP per capita (%)	
		2005	2013	2005	2013
**Afghanistan**	875.44	28.64	20.02	4.62	3.23
**Azerbaijan**	45.64	175.32	71.57	2.37	0.97
**Bangladesh**	910.09	45.29	21.43	6.06	2.87
**China**	1,752.85	183.09	69.32	2.96	1.12
**Eritrea**	98.98	26.08	10.09	5.17	2.00
**Guatemala**	132.69	21.50	10.40	0.64	0.31
**India**	8,522.64	81.22	26.04	5.45	1.75
**Indonesia**	1,137.93	46.41	21.81	1.30	0.61
**Korea, Democratic People's Republic**	82.09	72.59	25.41	14.35	5.02
**Kyrgyzstan**	24.97	62.42	27.13	5.38	2.34
**Lao People's Democratic Republic**	124.61	19.78	7.60	1.41	0.54
**Madagascar**	370.93	18.71	9.44	4.18	2.11
**Mexico**	212.37	382.68	93.97	3.93	0.96
**Myanmar**	327.07	35.70	17.92	3.12	1.57
**Nepal**	165.78	36.42	22.14	5.15	3.13
**Niger**	635.69	13.68	8.31	3.57	2.17
**Pakistan**	3,212.46	23.38	12.63	1.81	0.98
**Papua New Guinea**	96.55	29.03	13.10	1.33	0.60
**Peru**	57.94	174.37	46.57	2.65	0.71
**Philippines**	595.98	72.89	27.07	2.82	1.05
**Senegal**	165.98	26.17	11.95	2.54	1.16
**Somalia**	476.59	9.66	5.28	0.13	0.07
**Tajikistan**	107.30	29.31	17.54	4.81	2.88
**Turkmenistan**	46.83	69.66	20.26	1.07	0.31
**Uzbekistan**	241.88	37.58	16.95	2.19	0.99
**Yemen**	303.25	52.98	23.55	3.55	1.58
**Bolivia (Plurinational State of)**	81.97	23.81	7.50	0.92	0.29
**Brazil**	167.92	173.73	47.00	1.53	0.41
**Cambodia**	120.25	17.65	7.05	1.87	0.75
**Comoros**	13.03	50.80	42.64	6.12	5.13
**Egypt**	227.57	65.04	18.85	2.04	0.59
**Iraq**	265.05	114.60	58.90	1.78	0.91
**Morocco**	151.78	73.55	43.13	2.53	1.49
**Solomon Islands**	4.47	45.92	25.81	3.02	1.70
**Viet Nam**	214.59	85.53	35.54	5.36	2.23
**Benin**	191.87	14.16	5.83	1.88	0.77
**Burkina Faso**	390.63	20.65	9.14	3.26	1.44
**Burundi**	250.15	10.87	8.09	4.33	3.22
**Cameroon**	499.15	13.94	5.50	1.21	0.48
**Congo**	37.44	85.04	27.55	2.70	0.87
**Congo,**	1,770.31	10.24	5.14	3.76	1.89

Country	Total DALY averted (Thousands)	Cost per DALY averted (US$,2013)		Percentage of cost per DALY averted in GDP per capita (%)	
		2005	2013	2005	2013
**Democratic Republic**					
**Ethiopia**	1,568.40	12.24	7.06	2.60	1.50
**Gambia**	29.69	18.64	8.79	3.64	1.72
**Ghana**	352.92	16.87	7.29	1.05	0.45
**Guinea**	224.05	16.98	9.32	2.87	1.58
**Liberia**	64.60	19.14	11.66	4.54	2.77
**Malawi**	215.99	18.82	13.77	7.02	5.14
**Mali**	515.92	13.34	7.43	1.92	1.07
**Mauritania**	80.87	23.19	12.80	2.10	1.16
**Mozambique**	423.41	32.33	25.00	5.59	4.32
**Nigeria**	4,499.06	20.90	12.06	1.34	0.78
**Rwanda**	174.96	13.82	8.99	2.23	1.45
**Sierra Leone**	184.08	9.48	4.84	1.49	0.76
**Togo**	117.10	16.21	8.99	0.36	0.20
**Uganda**	654.10	15.51	9.13	2.83	1.67
**Zambia**	311.04	15.23	8.91	1.04	0.61
**Angola**	971.25	25.11	6.68	0.46	0.12
**Botswana**	9.75	253.83	84.47	3.53	1.17
**Central African Republic**	113.76	69.51	70.87	14.71	14.99
**Chad**	516.30	10.11	3.62	1.14	0.41
**Côte d'Ivoire**	391.80	26.75	12.21	2.15	0.98
**Djibouti**	11.01	27.90	9.54	2.63	0.90
**Equatorial Guinea**	14.30	353.62	112.66	1.47	0.47
**Gabon**	16.33	172.73	46.01	1.51	0.40
**Guinea-Bissau**	41.79	10.65	4.20	1.97	0.78
**Haiti**	172.73	10.52	5.01	1.36	0.65
**Kenya**	817.99	16.14	10.00	1.87	1.16
**Lesotho**	24.95	20.94	11.31	1.76	0.95
**Sao Tome and Principe**	2.46	26.25	17.38	1.87	1.24
**South Africa**	320.65	156.96	60.73	9.93	3.84
**Sudan**	772.94	43.67	22.32	1.43	0.73
**Swaziland**	15.91	36.47	20.27	4.18	2.32
**Tanzania, United Republic of**	634.76	25.09	18.85	4.37	3.28
**Zimbabwe**	238.01	11.37	6.71	1.44	0.85
**Total**	39,613.61				
**Median (IQR)**	212.37 (82.00-493.51)	26.17 (16.90-64.38)	12.80 (8.82-25.71)		

III. Effective access to care scenario

Based on data from published studies reporting effective access to care (product of geographic access, staff availability and medicine availability)

1. In countries without CCM in place, effective access rate for rural population is 9%
2. In countries with CCM in place, effective access rate for rural population is 30% in a typical setting

Supplementary 8 table: Total cost of pneumonia treatment in 2013 using effective access to care scenario

Delivery Levels	Total Cost for Pneumonia Treatment in 2013 (Billions, in US$ 2013)				
	2005 Guidelines		2013 Guidelines		2013/2005 %
	Total Cost	% OG Total	Total Cost	% RG Total	
**Community**	**1.25**	**55.7%**	**1.25**	**80.0%**	**100.1%**
**First Level Facility**	**0.11**	**4.9%**	**0.14**	**8.7%**	**123.9%**
**Hospital**	**0.88**	**39.4%**	**0.18**	**11.3%**	**20.0%**
**Total cost**	**2.24**	**100.0%**	**1.56**	**100.0%**	**69.7%**

Supplementary 9 table: Total cost of pneumonia treatment by HIV status using effective access to care scenario

Country	2005 Guidelines (Thousands)			2013 Guidelines(Thousands)			Total Cost Savings (Thousands)	2013 Guidelines	
	HIV+	HIV-	Total	HIV+	HIV-	Total		Total cost per capita	Proportion of national healthcare expenditure per capita
Afghanistan	2.37	22,983.10	22,985.46	2.45	19,476.64	19,479.09	3,506.38	0.64	1.14
Azerbaijan	27.23	7,353.17	7,380.40	36.68	4,145.55	4,182.23	3,198.17	0.44	0.12
Bangladesh	1.53	69,824.89	69,826.42	1.66	58,995.22	58,996.88	10,829.54	0.38	1.42
China	54.76	498,861.94	498,916.70	76.06	368,378.92	368,454.97	130,461.73	0.27	0.10
Eritrea	7.75	3,358.98	3,366.73	9.72	2,645.94	2,655.66	711.06	0.42	3.02
Guatemala	10.87	5,143.65	5,154.53	11.40	4,187.95	4,199.35	955.17	0.27	0.13
India	870.08	734,072.33	734,942.41	1,141.77	489,753.22	490,894.99	244,047.43	0.39	0.66
Indonesia	83.32	86,960.50	87,043.82	99.01	68,611.90	68,710.92	18,332.91	0.27	0.29
Korea, Democratic People's Republic	0.15	8,416.72	8,416.87	0.20	5,619.39	5,619.59	2,797.29	0.23	0.60
Kyrgyzstan	0.24	2,412.91	2,413.14	0.27	1,930.55	1,930.81	482.33	0.35	0.49
Lao People's Democratic Republic	4.85	3,263.79	3,268.64	5.69	2,443.38	2,449.07	819.57	0.36	0.98
Madagascar	29.18	10,445.32	10,474.50	30.36	8,628.49	8,658.85	1,815.65	0.38	1.99
Mexico	37.40	79,663.01	79,700.42	54.18	27,724.08	27,778.26	51,922.16	0.23	0.04
Myanmar	35.99	21,776.89	21,812.88	40.76	18,699.48	18,740.24	3,072.64	0.35	1.56
Nepal	6.56	12,832.62	12,839.19	6.98	11,840.68	11,847.65	991.53	0.43	1.29
Niger	35.32	10,360.75	10,396.07	36.08	8,907.97	8,944.05	1,452.02	0.50	2.49
Pakistan	12.63	93,620.30	93,632.93	14.28	74,519.50	74,533.78	19,099.16	0.41	1.38
Papua New Guinea	8.21	3,956.95	3,965.17	9.95	3,359.61	3,369.55	595.61	0.46	0.58
Peru	16.07	11,272.29	11,288.36	22.59	5,039.79	5,062.38	6,225.99	0.17	0.06
Philippines	2.35	49,581.11	49,583.46	2.94	32,050.96	32,053.90	17,529.56	0.33	0.34
Senegal	22.56	6,098.60	6,121.16	25.59	4,684.96	4,710.56	1,410.61	0.33	0.50
Somalia	27.87	5,510.87	5,538.74	27.92	4,333.33	4,361.24	1,177.49	0.42	10.76
Tajikistan	2.72	4,247.62	4,250.35	2.99	3,634.21	3,637.20	613.15	0.44	0.82
Turkmenistan	2.58	3,234.31	3,236.89	3.54	1,750.36	1,753.89	1,482.99	0.33	0.26

Country	2005 Guidelines (Thousands)			2013 Guidelines(Thousands)			Total Cost Savings (Thousands)	2013 Guidelines	
	HIV+	HIV-	Total	HIV+	HIV-	Total		Total cost per capita	Proportion of national healthcare expenditure per capita
Uzbekistan	14.94	13,076.41	13,091.35	17.86	10,312.87	10,330.73	2,760.62	0.36	0.40
Yemen	7.93	15,916.27	15,924.20	9.33	11,218.24	11,227.57	4,696.63	0.46	0.52
Bolivia (Plurinational State of)	2.83	1,359.93	1,362.76	3.56	401.53	405.09	957.67	0.04	0.03
Brazil	12.99	25,078.55	25,091.54	16.74	6,600.47	6,617.21	18,474.33	0.03	0.003
Cambodia	6.76	570.01	576.77	7.50	204.99	212.49	364.28	0.02	0.03
Comoros	0.09	228.38	228.46	0.09	168.13	168.22	60.24	0.26	0.61
Egypt	0.51	7,190.24	7,190.75	0.66	1,978.63	1,979.29	5,211.46	0.03	0.02
Iraq	215.74	20,889.61	21,105.35	269.60	9,550.93	9,820.53	11,284.82	0.32	0.10
Morocco	3.82	6,794.47	6,798.29	4.60	3,561.05	3,565.65	3,232.64	0.12	0.06
Solomon Islands	0.23	56.46	56.69	0.26	28.32	28.58	28.11	0.06	0.04
Viet Nam	27.65	6,806.54	6,834.19	32.11	2,571.91	2,604.02	4,230.16	0.03	0.03
Benin	27.17	4,074.22	4,101.40	29.94	3,089.34	3,119.28	982.12	0.30	0.82
Burkina Faso	65.22	9,399.75	9,464.97	69.14	7,212.77	7,281.91	2,183.06	0.43	1.16
Burundi	29.68	5,097.29	5,126.97	29.82	4,835.35	4,865.17	261.80	0.48	2.05
Cameroon	237.32	8,895.12	9,132.44	277.74	6,055.97	6,333.71	2,798.72	0.28	0.42
Congo	105.28	2,947.56	3,052.84	138.97	1,308.99	1,447.96	1,604.88	0.33	0.37
Congo, Democratic Republic	235.60	28,962.86	29,198.46	234.85	24,093.41	24,328.26	4,870.20	0.36	1.83
Ethiopia	233.11	42,589.05	42,822.16	239.52	39,175.95	39,415.46	3,406.69	0.42	2.52
Gambia	9.33	717.10	726.43	10.03	511.45	521.47	204.96	0.28	1.03
Ghana	79.06	9,149.47	9,228.53	93.56	6,892.37	6,985.93	2,242.60	0.27	0.36
Guinea	55.99	5,383.43	5,439.42	58.50	4,440.23	4,498.73	940.69	0.38	1.29
Liberia	11.71	2,166.24	2,177.95	11.79	1,858.42	1,870.22	307.73	0.30	0.55
Malawi	230.20	7,635.97	7,866.17	235.48	7,184.49	7,419.96	446.21	0.45	1.47
Mali	61.18	8,179.02	8,240.20	64.89	6,515.06	6,579.96	1,660.24	0.43	0.96
Mauritania	11.48	2,125.86	2,137.34	12.79	1,628.51	1,641.30	496.03	0.42	0.73
Mozambique	986.58	14,462.60	15,449.18	1,021.50	12,817.44	13,838.93	1,610.24	0.54	1.52

Country	2005 Guidelines (Thousands)			2013 Guidelines(Thousands)			Total Cost Savings (Thousands)	2013 Guidelines	
	HIV+	HIV-	Total	HIV+	HIV-	Total		Total cost per capita	Proportion of national healthcare expenditure per capita
**Nigeria**	2,853.71	97,968.95	100,822.65	3,268.98	71,792.26	75,061.24	25,761.41	0.43	0.54
**Rwanda**	27.64	5,268.18	5,295.82	29.25	4,899.86	4,929.11	366.71	0.42	0.67
**Sierra Leone**	24.06	2,595.81	2,619.88	24.64	2,104.92	2,129.56	490.31	0.35	0.51
**Togo**	60.84	2,864.08	2,924.91	64.05	2,381.95	2,446.00	478.91	0.36	0.80
**Uganda**	326.97	18,022.59	18,349.57	339.20	16,305.36	16,644.56	1,705.01	0.44	1.04
**Zambia**	405.28	6,179.11	6,584.39	467.22	4,988.60	5,455.82	1,128.57	0.38	0.43
**Angola**	370.15	14,963.65	15,333.79	508.21	3,625.88	4,134.09	11,199.70	0.19	0.10
**Botswana**	176.10	1,435.08	1,611.18	253.23	313.04	566.27	1,044.91	0.27	0.06
**Central African Republic**	240.03	3,282.44	3,522.47	242.58	3,350.56	3,593.14	-70.67	0.78	4.26
**Chad**	83.55	1,421.72	1,505.28	91.76	450.74	542.51	962.77	0.04	0.12
**Côte d'Ivoire**	314.77	5,520.43	5,835.20	354.98	2,327.69	2,682.67	3,152.53	0.13	0.17
**Djibouti**	7.97	235.11	243.08	9.27	74.38	83.65	159.43	0.10	0.09
**Equatorial Guinea**	231.29	2,040.21	2,271.50	348.73	420.26	769.00	1,502.51	0.96	0.08
**Gabon**	111.23	2,354.94	2,466.17	161.98	514.65	676.63	1,789.54	0.39	0.11
**Guinea-Bissau**	19.78	198.20	217.98	21.02	65.59	86.61	131.36	0.05	0.14
**Haiti**	31.67	1,014.95	1,046.62	33.96	465.75	499.71	546.91	0.05	0.08
**Kenya**	328.10	3,740.01	4,068.11	353.21	2,179.75	2,532.97	1,535.14	0.06	0.16
**Lesotho**	57.29	120.85	178.14	65.06	34.75	99.80	78.34	0.05	0.03
**Sao Tome and Principe**	1.11	41.49	42.60	1.25	27.03	28.27	14.33	0.15	0.12
**South Africa**	5,147.07	27,775.63	32,922.70	7,346.12	6,251.52	13,597.63	19,325.06	0.24	0.04
**Sudan**	45.52	12,112.82	12,158.34	51.23	6,164.45	6,215.68	5,942.66	0.16	0.16
**Swaziland**	47.90	116.53	164.43	62.22	34.95	97.18	67.26	0.07	0.03
**Tanzania, United Republic of**	414.45	4,891.70	5,306.15	429.12	3,566.29	3,995.41	1,310.74	0.08	0.22
**Zimbabwe**	313.32	880.88	1,194.20	335.42	381.84	717.26	476.95	0.05	0.13
**Total**	15,614.82	2,220,050.34	2,235,665.16	19,416.61	1,538,300.92	1,557,717.53	677,947.63		

Supplementary 10 table: Total DALYs averted and cost-effectiveness of implementing 2013 guidelines using effective access to care scenario

Country	Total DALY averted (Thousands)	Cost per DALY averted (US$,2013)		Percentage of cost per DALY averted in GDP per capita (%)	
		2005	2013	2005	2013
Afghanistan	875.44	26.26	22.25	4.24	3.59
Azerbaijan	45.64	161.70	91.63	2.19	1.24
Bangladesh	910.09	76.72	64.83	10.27	8.67
China	1,752.85	284.63	210.20	4.60	3.40
Eritrea	98.98	34.01	26.83	6.74	5.32
Guatemala	132.69	38.85	31.65	1.16	0.94
India	8,522.64	86.23	57.60	5.79	3.87
Indonesia	1,137.93	76.49	60.38	2.15	1.70
Korea, Democratic People's Republic	82.09	102.53	68.46	20.26	13.53
Kyrgyzstan	24.97	96.65	77.33	8.33	6.67
Lao People's Democratic Republic	124.61	26.23	19.65	1.87	1.40
Madagascar	370.93	28.24	23.34	6.31	5.22
Mexico	212.37	375.29	130.80	3.85	1.34
Myanmar	327.07	66.69	57.30	5.83	5.01
Nepal	165.78	77.45	71.47	10.96	10.11
Niger	635.69	16.35	14.07	4.27	3.68
Pakistan	3,212.46	29.15	23.20	2.26	1.80
Papua New Guinea	96.55	41.07	34.90	1.88	1.60
Peru	57.94	194.84	87.38	2.97	1.33
Philippines	595.98	83.20	53.78	3.22	2.08
Senegal	165.98	36.88	28.38	3.57	2.75
Somalia	476.59	11.62	9.15	0.15	0.12
Tajikistan	107.30	39.61	33.90	6.51	5.57
Turkmenistan	46.83	69.12	37.45	1.06	0.58
Uzbekistan	241.88	54.12	42.71	3.15	2.49
Yemen	303.25	52.51	37.02	3.51	2.48
Bolivia (Plurinational State of)	81.97	16.62	5.23	0.65	0.20
Brazil	167.92	149.42	40.43	1.32	0.36
Cambodia	120.25	4.80	1.92	0.51	0.20
Comoros	13.03	17.53	14.72	2.11	1.77
Egypt	227.57	31.60	9.16	0.99	0.29
Iraq	265.05	79.63	40.93	1.23	0.63
Morocco	151.78	44.79	26.27	1.54	0.91
Solomon Islands	4.47	12.68	7.13	0.84	0.47
Viet Nam	214.59	31.85	13.24	2.00	0.83
Benin	191.87	21.38	16.26	2.84	2.16
Burkina Faso	390.63	24.23	18.64	3.82	2.94
Burundi	250.15	20.50	19.45	8.17	7.75
Cameroon	499.15	18.30	12.69	1.59	1.10
Congo	37.44	81.53	38.67	2.59	1.23
Congo,	1,770.31	16.49	13.74	6.06	5.05

Country	Total DALY averted (Thousands)	Cost per DALY averted (US$,2013)		Percentage of cost per DALY averted in GDP per capita (%)	
		2005	2013	2005	2013
Democratic Republic					
Ethiopia	1,568.40	27.30	25.13	5.81	5.34
Gambia	29.69	24.46	17.56	4.78	3.43
Ghana	352.92	26.15	19.79	1.63	1.23
Guinea	224.05	24.28	20.08	4.11	3.40
Liberia	64.60	33.72	28.95	8.00	6.87
Malawi	215.99	36.42	34.35	13.59	12.82
Mali	515.92	15.97	12.75	2.30	1.84
Mauritania	80.87	26.43	20.29	2.39	1.83
Mozambique	423.41	36.49	32.68	6.30	5.65
Nigeria	4,499.06	22.41	16.68	1.44	1.07
Rwanda	174.96	30.27	28.17	4.88	4.54
Sierra Leone	184.08	14.23	11.57	2.24	1.82
Togo	117.10	24.98	20.89	0.56	0.46
Uganda	654.10	28.05	25.45	5.13	4.65
Zambia	311.04	21.17	17.54	1.44	1.19
Angola	971.25	15.79	4.20	0.29	0.08
Botswana	9.75	165.25	54.99	2.30	0.76
Central African Republic	113.76	30.97	31.57	6.55	6.68
Chad	516.30	2.92	1.04	0.33	0.12
Côte d'Ivoire	391.80	14.89	6.80	1.20	0.55
Djibouti	11.01	22.08	7.54	2.08	0.71
Equatorial Guinea	14.30	158.86	50.61	0.66	0.21
Gabon	16.33	151.05	40.23	1.32	0.35
Guinea-Bissau	41.79	5.22	2.06	0.97	0.38
Haiti	172.73	6.06	2.89	0.79	0.37
Kenya	817.99	4.97	3.08	0.58	0.36
Lesotho	24.95	7.14	3.86	0.60	0.32
Sao Tome and Principe	2.46	17.34	11.48	1.24	0.82
South Africa	320.65	102.68	39.73	6.50	2.51
Sudan	772.94	15.73	8.04	0.52	0.26
Swaziland	15.91	10.33	5.74	1.18	0.66
Tanzania, United Republic of	634.76	8.36	6.28	1.46	1.09
Zimbabwe	238.01	5.02	2.96	0.64	0.38
Total	39,613.61				
Median (IQR)	212.37 (82.00-493.51)	28.05 (16.80-68.51)	22.25 (11.85-39.46)		

IV. New (unpublished) data for proportion of pneumonia signs in HIV+ children

In high HIV prevalence setting (Malawi) the proportion of pneumonia cases with fast breathing only/ chest indrawing/ dangerous signs has been reported 85%, 1.5%, 13.5% (Tim Colbourn, personal communication). These are program data reported by health workers trained in iCCM. We applied this split to the estimated number of HIV-infected children with pneumonia reported in the literature. Split for HIV uninfected children remained as before- i.e. 85%, 13%, 2%. All other assumptions remain the same as the main model.

Supplementary 11 table: Total cost of pneumonia treatment in 2013 using unpublished data on proportion of clinical signs in children with pneumonia in a high HIV burden setting

	Total Cost for Pneumonia Treatments in 2013 (Billions, in US$ 2013)				
**Delivery Levels**	**2005 Guidelines (OG)**		**2013 Guidelines(RG)**		**2013/2005 %**
	**Total Cost**	**%Total**	**Total Cost**	**%Total**	
**Community**	1.25	42.3%	1.25	70.7%	100.3%
**First Level Facility**	0.18	6.1%	0.23	12.8%	126.0%
**Hospital**	1.52	51.6%	0.29	16.5%	19.1%
**Total cost**	2.95	100%	1.77	100.0%	60.0%

Supplementary 12 table: Total cost of pneumonia treatment by HIV status using unpublished data on proportion of clinical signs in children with pneumonia in high HIV burden settings

Country	2005 Guidelines (Thousands)			2013 Guidelines(Thousands)			Total Cost Savings (Thousands)	2013 Guidelines	
	HIV+	HIV-	Total	HIV+	HIV-	Total		Total cost per capita	Proportion of national healthcare expenditure per capita
**Afghanistan**	6.21	34,135.63	34,141.84	5.24	26,589.69	26,594.93	7,546.92	0.87	1.56
**Azerbaijan**	54.66	9,656.73	9,711.39	53.97	4,907.24	4,961.21	4,750.18	0.53	0.15
**Bangladesh**	4.29	84,736.32	84,740.60	3.53	63,016.38	63,019.91	21,720.69	0.40	1.52
**China**	121.39	586,413.86	586,535.25	118.11	386,959.53	387,077.64	199,457.61	0.28	0.10
**Eritrea**	24.65	4,497.40	4,522.05	22.35	2,910.33	2,932.68	1,589.37	0.46	3.33
**Guatemala**	23.40	5,851.12	5,874.51	18.78	4,377.21	4,395.99	1,478.52	0.28	0.13
**India**	2,412.75	1,024,440.35	1,026,853.11	2,256.05	553,627.25	555,883.30	470,969.81	0.44	0.75
**Indonesia**	176.69	100,469.43	100,646.12	156.11	72,455.25	72,611.36	28,034.76	0.29	0.31
**Korea, Democratic People's Republic**	0.30	9,792.33	9,792.63	0.28	5,919.03	5,919.31	3,873.32	0.24	0.63
**Kyrgyzstan**	0.60	2,949.80	2,950.40	0.52	2,068.64	2,069.16	881.24	0.37	0.52
**Lao People's Democratic Republic**	12.73	4,182.93	4,195.66	11.02	2,662.95	2,673.97	1,521.69	0.39	1.08
**Madagascar**	75.40	12,884.32	12,959.72	60.71	9,443.03	9,503.74	3,455.98	0.41	2.18
**Mexico**	64.24	91,613.44	91,677.68	64.51	30,279.36	30,343.87	61,333.81	0.25	0.04
**Myanmar**	95.27	25,527.24	25,622.51	80.80	19,704.29	19,785.08	5,837.42	0.37	1.65
**Nepal**	21.35	14,976.83	14,998.18	17.49	12,608.62	12,626.11	2,372.07	0.45	1.38
**Niger**	108.11	14,302.62	14,410.73	87.78	10,883.19	10,970.97	3,439.76	0.62	3.06
**Pakistan**	29.93	120,173.52	120,203.45	26.15	85,654.55	85,680.70	34,522.76	0.47	1.58
**Papua New Guinea**	29.82	5,268.41	5,298.22	26.56	3,725.83	3,752.39	1,545.84	0.51	0.65
**Peru**	27.81	12,763.31	12,791.12	27.26	5,350.99	5,378.25	7,412.87	0.18	0.06
**Philippines**	5.13	62,967.52	62,972.64	4.69	35,656.88	35,661.57	27,311.07	0.36	0.38
**Senegal**	52.05	7,458.85	7,510.90	44.53	5,093.66	5,138.19	2,372.72	0.36	0.54
**Somalia**	65.29	7,093.02	7,158.31	51.83	5,005.91	5,057.74	2,100.57	0.48	12.48
**Tajikistan**	7.23	5,479.34	5,486.57	6.23	4,216.14	4,222.36	1,264.21	0.51	0.95

Country	2005 Guidelines (Thousands)			2013 Guidelines(Thousands)			Total Cost Savings (Thousands)	2013 Guidelines	
	HIV+	HIV-	Total	HIV+	HIV-	Total		Total cost per capita	Proportion of national healthcare expenditure per capita
**Turkmenistan**	5.80	4,301.07	4,306.87	5.61	1,986.10	1,991.71	2,315.16	0.38	0.29
**Uzbekistan**	37.41	16,226.31	16,263.72	33.21	11,232.11	11,265.32	4,998.40	0.39	0.44
**Yemen**	20.33	22,461.41	22,481.74	18.06	13,533.47	13,551.53	8,930.21	0.56	0.63
**Bolivia (Plurinational State of)**	5.72	1,948.07	1,953.78	5.26	577.56	582.82	1,370.96	0.06	0.05
**Brazil**	21.54	29,158.90	29,180.44	20.04	7,847.54	7,867.59	21,312.85	0.04	0.004
**Cambodia**	34.32	2,097.41	2,131.73	29.18	754.28	783.47	1,348.26	0.06	0.11
**Comoros**	0.30	661.70	662.00	0.27	487.15	487.42	174.58	0.76	1.78
**Egypt**	1.49	14,799.49	14,800.98	1.39	4,072.57	4,073.96	10,727.03	0.05	0.04
**Iraq**	412.44	30,063.38	30,475.82	395.50	13,745.27	14,140.77	16,335.05	0.46	0.14
**Morocco**	8.11	11,156.99	11,165.11	7.71	5,847.48	5,855.20	5,309.91	0.20	0.11
**Solomon Islands**	1.09	204.44	205.53	0.98	102.53	103.51	102.02	0.21	0.15
**Viet Nam**	101.84	18,278.60	18,380.45	90.05	6,906.74	6,996.79	11,383.66	0.08	0.09
**Benin**	62.63	4,878.91	4,941.54	51.77	3,275.94	3,327.71	1,613.83	0.32	0.88
**Burkina Faso**	185.04	12,763.14	12,948.19	150.42	8,259.09	8,409.51	4,538.67	0.50	1.33
**Burundi**	101.02	6,156.61	6,257.63	81.89	5,460.68	5,542.57	715.06	0.55	2.33
**Cameroon**	509.17	10,737.84	11,247.01	438.77	6,466.27	6,905.04	4,341.96	0.31	0.45
**Congo**	202.51	3,670.67	3,873.18	190.72	1,473.04	1,663.75	2,209.43	0.37	0.43
**Congo, Democratic Republic**	601.89	34,908.51	35,510.40	467.42	25,889.25	26,356.67	9,153.73	0.39	1.98
**Ethiopia**	781.87	48,956.23	49,738.10	616.02	40,809.98	41,426.00	8,312.10	0.44	2.65
**Gambia**	18.20	846.88	865.09	15.04	553.47	568.50	296.58	0.31	1.12
**Ghana**	168.34	10,671.04	10,839.38	147.01	7,267.84	7,414.86	3,424.52	0.29	0.38
**Guinea**	136.73	6,646.82	6,783.55	111.40	4,925.69	5,037.09	1,746.46	0.43	1.44
**Liberia**	24.35	2,465.72	2,490.07	19.42	1,982.63	2,002.05	488.02	0.32	0.59
**Malawi**	739.44	8,873.99	9,613.44	598.20	7,771.98	8,370.18	1,243.25	0.51	1.65
**Mali**	147.36	10,637.06	10,784.42	122.55	7,580.94	7,703.49	3,080.93	0.50	1.13
**Mauritania**	25.29	2,740.12	2,765.41	21.95	1,897.56	1,919.51	845.91	0.49	0.86

Country	2005 Guidelines (Thousands)			2013 Guidelines(Thousands)			Total Cost Savings (Thousands)	2013 Guidelines	
	HIV+	HIV-	Total	HIV+	HIV-	Total		Total cost per capita	Proportion of national healthcare expenditure per capita
**Mozambique**	2,291.26	18,813.27	21,104.53	1,944.45	15,639.12	17,583.57	3,520.95	0.68	1.93
**Nigeria**	5,688.48	123,748.56	129,437.04	5,074.64	83,315.73	88,390.36	41,046.67	0.51	0.64
**Rwanda**	85.87	6,052.42	6,138.29	70.45	5,203.11	5,273.56	864.73	0.45	0.71
**Sierra Leone**	57.12	3,140.00	3,197.12	45.47	2,285.72	2,331.20	865.92	0.38	0.56
**Togo**	145.54	3,433.18	3,578.73	118.65	2,581.48	2,700.13	878.59	0.40	0.88
**Uganda**	1,104.52	21,666.61	22,771.13	880.49	17,467.48	18,347.97	4,423.15	0.49	1.15
**Zambia**	965.35	7,479.67	8,445.02	837.37	5,405.86	6,243.24	2,201.78	0.43	0.49
**Angola**	848.87	23,802.80	24,651.67	823.20	5,767.72	6,590.92	18,060.75	0.28	0.15
**Botswana**	393.85	2,204.32	2,598.17	393.13	480.84	873.97	1,724.20	0.28	0.06
**Central African Republic**	593.11	7,368.55	7,961.66	555.61	7,521.47	8,077.08	-115.42	1.74	9.54
**Chad**	406.55	4,931.14	5,337.69	338.85	1,563.37	1,902.22	3,435.47	0.14	0.40
**Côte d'Ivoire**	773.93	9,913.63	10,687.56	670.01	4,180.08	4,850.09	5,837.47	0.23	0.29
**Djibouti**	14.15	297.13	311.28	12.31	94.00	106.31	204.97	0.12	0.11
**Equatorial Guinea**	755.10	4,541.29	5,296.39	778.27	935.46	1,713.73	3,582.66	1.36	0.11
**Gabon**	185.37	2,693.01	2,878.37	186.77	588.53	775.30	2,103.07	0.37	0.10
**Guinea-Bissau**	56.48	404.74	461.21	46.04	133.95	179.99	281.23	0.10	0.27
**Haiti**	74.67	1,761.58	1,836.25	62.58	808.36	870.94	965.30	0.08	0.14
**Kenya**	1,397.86	12,138.81	13,536.67	1,205.03	7,074.75	8,279.78	5,256.89	0.18	0.50
**Lesotho**	237.46	354.51	591.97	201.90	101.93	303.83	288.14	0.11	0.08
**Sao Tome and Principe**	2.15	62.80	64.96	1.95	40.91	42.86	22.09	0.22	0.19
**South Africa**	11,428.06	42,460.96	53,889.02	11,361.75	9,556.77	20,918.52	32,970.50	0.23	0.03
**Sudan**	168.97	33,629.99	33,798.95	148.65	17,114.97	17,263.62	16,535.33	0.45	0.44
**Swaziland**	239.27	411.29	650.57	225.34	123.37	348.71	301.86	0.17	0.06
**Tanzania, United Republic of**	1,564.63	14,681.66	16,246.29	1,351.26	10,703.65	12,054.91	4,191.38	0.24	0.65
**Zimbabwe**	970.42	1,996.04	2,966.46	808.62	865.22	1,673.84	1,292.61	0.11	0.27
**Total**	38,222.58	2,911,933.58	2,950,156.16	34,927.15	1,743,376.97	1,778,304.12	1,171,852.04		

Supplementary 13 table: Total DALYs averted and cost-effectiveness of implementing 2013 guidelines using unpublished data on proportion of clinical signs in children with pneumonia in high HIV burden settings

Country	Total DALY averted (Thousands)	Cost per DALY averted (US$,2013)		Percentage of cost per DALY averted in GDP per capita (%)	
		2005	2013	2005	2013
**Afghanistan**	875.44	39.00	30.38	6.29	4.90
**Azerbaijan**	45.64	212.77	108.70	2.88	1.47
**Bangladesh**	910.09	93.11	69.25	12.46	9.27
**China**	1,752.85	334.62	220.83	5.41	3.57
**Eritrea**	98.98	45.69	29.63	9.06	5.88
**Guatemala**	132.69	44.27	33.13	1.32	0.99
**India**	8,522.64	120.49	65.22	8.09	4.38
**Indonesia**	1,137.93	88.45	63.81	2.49	1.79
**Korea, Democratic People's Republic**	82.09	119.29	72.11	23.58	14.25
**Kyrgyzstan**	24.97	118.16	82.87	10.19	7.15
**Lao People's Democratic Republic**	124.61	33.67	21.46	2.41	1.53
**Madagascar**	370.93	34.94	25.62	7.81	5.73
**Mexico**	212.37	431.68	142.88	4.43	1.47
**Myanmar**	327.07	78.34	60.49	6.85	5.29
**Nepal**	165.78	90.47	76.16	12.80	10.78
**Niger**	635.69	22.67	17.26	5.92	4.51
**Pakistan**	3,212.46	37.42	26.67	2.90	2.07
**Papua New Guinea**	96.55	54.88	38.87	2.51	1.78
**Peru**	57.94	220.77	92.83	3.36	1.41
**Philippines**	595.98	105.66	59.84	4.08	2.31
**Senegal**	165.98	45.25	30.96	4.39	3.00
**Somalia**	476.59	15.02	10.61	0.20	0.14
**Tajikistan**	107.30	51.13	39.35	8.40	6.46
**Turkmenistan**	46.83	91.97	42.53	1.41	0.65
**Uzbekistan**	241.88	67.24	46.57	3.92	2.71
**Yemen**	303.25	74.13	44.69	4.96	2.99
**Bolivia (Plurinational State of)**	81.97	23.83	7.50	0.93	0.29
**Brazil**	167.92	173.77	47.01	1.53	0.41
**Cambodia**	120.25	17.73	7.07	1.87	0.75
**Comoros**	13.03	50.81	42.64	6.12	5.13
**Egypt**	227.57	65.04	18.85	2.04	0.59
**Iraq**	265.05	114.98	58.93	1.78	0.91
**Morocco**	151.78	73.56	43.14	2.53	1.49
**Solomon Islands**	4.47	45.97	25.81	3.03	1.70
**Viet Nam**	214.59	85.65	35.56	5.37	2.23
**Benin**	191.87	25.75	17.34	3.43	2.31
**Burkina Faso**	390.63	33.15	21.53	5.23	3.39
**Burundi**	250.15	25.02	22.16	9.97	8.83
**Cameroon**	499.15	22.53	13.83	1.96	1.20
**Congo**	37.44	103.44	44.43	3.28	1.41
**Congo, Democratic**	1,770.31	20.06	14.89	7.38	5.47

Country	Total DALY averted (Thousands)	Cost per DALY averted (US$,2013)		Percentage of cost per DALY averted in GDP per capita (%)	
		2005	2013	2005	2013
**Republic**					
**Ethiopia**	1,568.40	31.71	26.41	6.74	5.62
**Gambia**	29.69	29.13	19.15	5.69	3.74
**Ghana**	352.92	30.71	21.01	1.91	1.31
**Guinea**	224.05	30.28	22.48	5.12	3.80
**Liberia**	64.60	38.55	30.99	9.14	7.35
**Malawi**	215.99	44.51	38.75	16.60	14.46
**Mali**	515.92	20.90	14.93	3.01	2.15
**Mauritania**	80.87	34.19	23.74	3.09	2.15
**Mozambique**	423.41	49.84	41.53	8.61	7.17
**Nigeria**	4,499.06	28.77	19.65	1.85	1.26
**Rwanda**	174.96	35.08	30.14	5.66	4.86
**Sierra Leone**	184.08	17.37	12.66	2.74	1.99
**Togo**	117.10	30.56	23.06	0.68	0.51
**Uganda**	654.10	34.81	28.05	6.36	5.13
**Zambia**	311.04	27.15	20.07	1.85	1.37
**Angola**	971.25	25.38	6.18	0.46	0.11
**Botswana**	9.75	266.48	57.62	3.71	0.80
**Central African Republic**	113.76	69.99	70.77	14.81	14.97
**Chad**	516.30	10.34	3.50	1.17	0.40
**Côte d'Ivoire**	391.80	27.28	11.81	2.19	0.95
**Djibouti**	11.01	28.27	9.21	2.66	0.87
**Equatorial Guinea**	14.30	370.42	72.24	1.54	0.30
**Gabon**	16.33	176.30	38.15	1.54	0.33
**Guinea-Bissau**	41.79	11.04	4.07	2.05	0.75
**Haiti**	172.73	10.63	4.96	1.38	0.64
**Kenya**	817.99	16.55	9.78	1.91	1.13
**Lesotho**	24.95	23.73	9.32	1.99	0.78
**Sao Tome and Principe**	2.46	26.43	17.20	1.89	1.23
**South Africa**	320.65	168.06	37.57	10.64	2.38
**Sudan**	772.94	43.73	22.27	1.44	0.73
**Swaziland**	15.91	40.88	13.27	4.69	1.52
**Tanzania, United Republic of**	634.76	25.59	18.70	4.46	3.26
**Zimbabwe**	238.01	12.46	6.26	1.58	0.79
**Total**	39,613.61	74.47	44.67		
**Median**	212.37	39.00	26.67		
**(IQR)**	(82.00-493.51)	(26.61-87.75)	(17.28-44.62)		

V. High cost less effective scenario

High cost less effective scenario assumptions:

1. CCM Coverage: 100% coverage of rural population in countries with CCM
2. Unit cost of medicine changed based on review by Zhang et al 2015 (in press)
3. Mortality reduction with 2013 guidelines: 36%, 2005 guidelines remain 70%.

All other assumptions are the same as the main model.

Supplementary 14 table: Total cost of pneumonia treatment in a high cost less effective scenario

Delivery Levels	Total Cost for Pneumonia Treatment in 2013 (Billions, in US$ 2013)				
	2005 Guidelines		2013 Guidelines		2013/2005 %
	Total Cost	%Total	Total Cost	%Total	
**Community**	2.52	59.5%	2.51	83.3%	99.4%
**First Level Facility**	0.15	3.5%	0.17	5.8%	117.2%
**Hospital**	1.57	37.0%	0.33	10.9%	21.0%
**Total cost**	4.24	100%	3.01	100.0%	71.0%

Supplementary 15 table: Total cost of pneumonia treatment at national level by HIV status in a high cost less effective scenario

Country	2005 Guidelines (Thousands)			2013 Guidelines(Thousands)			Total Cost Savings (Thousands)	2013 Guidelines	
	HIV+	HIV-	Total	HIV+	HIV-	Total		Total cost per capita	Proportion of national healthcare expenditure per capita
**Afghanistan**	4.79	41,353.93	41,358.72	4.98	32,271.29	32,276.27	9,082.44	1.06	1.89
**Azerbaijan**	39.61	11,549.23	11,588.85	53.66	6,693.26	6,746.92	4,841.93	0.72	0.20
**Bangladesh**	3.67	141,956.16	141,959.83	3.96	117,337.27	117,341.22	24,618.61	0.75	2.82
**China**	89.67	925,232.53	925,322.21	122.36	720,361.46	720,483.83	204,838.38	0.52	0.19
**Eritrea**	19.43	7,012.79	7,032.22	23.88	5,325.57	5,349.45	1,682.77	0.84	6.08
**Guatemala**	20.64	9,894.60	9,915.24	21.54	8,188.10	8,209.65	1,705.59	0.53	0.25
**India**	1,853.51	1,466,513.18	1,468,366.70	2,381.22	973,976.43	976,357.66	492,009.04	0.78	1.32
**Indonesia**	143.61	162,156.09	162,299.70	168.01	131,798.36	131,966.38	30,333.32	0.53	0.56
**Korea, Democratic People's Republic**	0.23	14,801.09	14,801.32	0.30	10,760.73	10,761.03	4,040.29	0.43	1.15
**Kyrgyzstan**	0.50	4,783.63	4,784.14	0.57	3,809.90	3,810.47	973.66	0.69	0.96
**Lao People's Democratic Republic**	10.51	6,499.48	6,509.99	12.11	4,838.86	4,850.96	1,659.03	0.72	1.95
**Madagascar**	65.36	20,781.75	20,847.11	67.89	16,767.74	16,835.62	4,011.49	0.73	3.87
**Mexico**	45.88	105,690.21	105,736.10	65.74	43,507.76	43,573.50	62,162.60	0.36	0.06
**Myanmar**	79.71	43,523.94	43,603.64	89.04	37,059.04	37,148.07	6,455.57	0.70	3.09
**Nepal**	17.91	26,244.24	26,262.15	18.97	23,515.89	23,534.86	2,727.29	0.85	2.57
**Niger**	88.48	20,833.71	20,922.19	90.66	16,739.41	16,830.06	4,092.12	0.94	4.69
**Pakistan**	23.92	174,406.13	174,430.05	26.98	136,168.76	136,195.73	38,234.32	0.75	2.52
**Papua New Guinea**	23.51	8,413.58	8,437.08	28.05	6,752.68	6,780.73	1,656.35	0.93	1.17
**Peru**	20.40	16,362.31	16,382.71	28.18	8,773.92	8,802.10	7,580.61	0.29	0.10
**Philippines**	4.03	88,615.51	88,619.54	4.96	59,653.38	59,658.34	28,961.20	0.61	0.63
**Senegal**	43.30	11,593.75	11,637.05	48.54	8,972.44	9,020.97	2,616.08	0.64	0.95
**Somalia**	56.99	10,422.14	10,479.13	57.33	7,903.28	7,960.61	2,518.52	0.76	19.65
**Tajikistan**	5.71	8,204.51	8,210.22	6.29	6,779.03	6,785.32	1,424.90	0.83	1.53
**Turkmenistan**	4.31	5,680.81	5,685.12	5.81	3,296.20	3,302.01	2,383.11	0.63	0.49

Country	2005 Guidelines (Thousands)			2013 Guidelines(Thousands)			Total Cost Savings (Thousands)	2013 Guidelines	
	HIV+	HIV-	Total	HIV+	HIV-	Total		Total cost per capita	Proportion of national healthcare expenditure per capita
**Uzbekistan**	29.98	25,395.01	25,424.99	35.34	19,996.99	20,032.33	5,392.65	0.69	0.78
**Yemen**	16.06	30,290.64	30,306.70	18.77	20,594.14	20,612.91	9,693.79	0.84	0.96
**Bolivia (Plurinational State of)**	4.59	2,063.32	2,067.90	5.64	615.55	621.19	1,446.71	0.06	0.05
**Brazil**	16.95	30,751.76	30,768.72	21.35	8,372.61	8,393.97	22,374.75	0.04	0.004
**Cambodia**	29.91	2,325.99	2,355.90	32.75	829.63	862.38	1,493.52	0.06	0.12
**Comoros**	0.28	695.00	695.27	0.29	498.13	498.42	196.86	0.77	1.81
**Egypt**	1.18	15,579.34	15,580.52	1.48	4,329.63	4,331.11	11,249.40	0.06	0.04
**Iraq**	334.43	31,006.42	31,340.85	412.44	14,056.13	14,468.57	16,872.28	0.47	0.14
**Morocco**	6.77	11,527.88	11,534.65	8.07	5,969.74	5,977.81	5,556.84	0.20	0.11
**Solomon Islands**	0.94	217.12	218.06	1.06	106.71	107.77	110.29	0.21	0.16
**Viet Nam**	86.43	19,745.62	19,832.05	98.67	7,390.32	7,488.99	12,343.06	0.09	0.09
**Benin**	53.98	7,896.64	7,950.62	58.70	6,091.59	6,150.29	1,800.33	0.60	1.62
**Burkina Faso**	158.95	19,296.58	19,455.54	167.71	14,107.88	14,275.59	5,179.95	0.84	2.27
**Burundi**	79.42	10,241.17	10,320.58	80.17	9,384.00	9,464.17	856.42	0.93	3.99
**Cameroon**	424.31	16,442.50	16,866.81	486.61	11,768.36	12,254.97	4,611.84	0.55	0.81
**Congo**	155.34	4,570.15	4,725.49	200.84	2,278.13	2,478.97	2,246.52	0.56	0.64
**Congo, Democratic Republic**	539.44	58,209.59	58,749.03	540.63	47,401.89	47,942.52	10,806.51	0.71	3.60
**Ethiopia**	692.27	88,359.51	89,051.78	710.79	78,813.55	79,524.34	9,527.45	0.85	5.09
**Gambia**	15.75	1,270.27	1,286.02	16.80	935.08	951.88	334.14	0.51	1.88
**Ghana**	138.53	17,060.18	17,198.70	160.85	13,358.45	13,519.30	3,679.40	0.52	0.70
**Guinea**	116.96	10,434.55	10,551.51	122.03	8,428.03	8,550.06	2,001.45	0.73	2.45
**Liberia**	21.45	4,081.57	4,103.02	21.67	3,501.68	3,523.35	579.67	0.57	1.03
**Malawi**	608.95	15,428.66	16,037.61	624.58	14,114.16	14,738.74	1,298.87	0.90	2.91
**Mali**	122.94	15,410.62	15,533.56	130.26	11,877.86	12,008.11	3,525.45	0.78	1.76
**Mauritania**	20.47	3,798.48	3,818.95	22.76	2,857.39	2,880.15	938.80	0.74	1.28
**Mozambique**	1,795.55	25,845.05	27,640.59	1,869.72	22,021.97	23,891.69	3,748.91	0.92	2.63

Country	2005 Guidelines (Thousands)			2013 Guidelines(Thousands)			Total Cost Savings (Thousands)	2013 Guidelines	
	HIV+	HIV-	Total	HIV+	HIV-	Total		Total cost per capita	Proportion of national healthcare expenditure per capita
**Nigeria**	4,545.67	163,610.14	168,155.80	5,200.30	119,295.14	124,495.44	43,660.37	0.72	0.90
**Rwanda**	71.63	10,673.84	10,745.47	75.64	9,691.49	9,767.13	978.34	0.83	1.32
**Sierra Leone**	50.23	5,062.17	5,112.40	51.46	4,055.25	4,106.71	1,005.69	0.67	0.98
**Togo**	125.19	5,566.03	5,691.22	131.40	4,579.61	4,711.01	980.21	0.69	1.54
**Uganda**	950.05	37,474.71	38,424.76	972.01	32,586.46	33,558.48	4,866.29	0.89	2.11
**Zambia**	786.80	11,860.99	12,647.79	891.27	9,584.90	10,476.17	2,171.62	0.72	0.83
**Angola**	635.97	24,583.14	25,219.12	856.57	6,024.95	6,881.52	18,337.60	0.32	0.17
**Botswana**	283.79	2,246.15	2,529.95	402.54	494.63	897.17	1,632.77	0.42	0.10
**Central African Republic**	574.13	7,560.54	8,134.67	580.58	7,584.76	8,165.34	-30.66	1.77	9.67
**Chad**	355.45	5,570.50	5,925.95	385.29	1,774.13	2,159.42	3,766.53	0.17	0.48
**Côte d'Ivoire**	669.09	10,846.73	11,515.81	743.44	4,487.67	5,231.11	6,284.71	0.26	0.32
**Djibouti**	11.96	325.79	337.75	13.64	103.45	117.09	220.66	0.13	0.13
**Equatorial Guinea**	521.30	4,561.92	5,083.22	782.84	942.26	1,725.10	3,358.11	2.15	0.17
**Gabon**	132.09	2,732.22	2,864.31	190.23	601.46	791.69	2,072.62	0.46	0.13
**Guinea-Bissau**	50.33	464.76	515.09	53.06	153.73	206.79	308.29	0.12	0.32
**Haiti**	66.57	1,965.85	2,032.42	70.79	875.70	946.49	1,085.94	0.09	0.16
**Kenya**	1,251.93	13,210.48	14,462.41	1,337.33	7,428.01	8,765.34	5,697.07	0.20	0.54
**Lesotho**	203.37	395.33	598.70	226.89	115.39	342.27	256.43	0.16	0.11
**Sao Tome and Principe**	1.89	66.22	68.12	2.10	42.04	44.14	23.98	0.23	0.19
**South Africa**	8,288.46	43,342.47	51,630.93	11,658.89	9,847.35	21,506.24	30,124.69	0.38	0.06
**Sudan**	146.87	36,283.41	36,430.28	163.14	17,989.64	18,152.78	18,277.51	0.48	0.46
**Swaziland**	187.34	430.54	617.88	238.27	129.71	367.98	249.90	0.28	0.11
**Tanzania, United Republic of**	1,445.94	15,843.48	17,289.42	1,494.17	11,086.63	12,580.80	4,708.63	0.25	0.68
**Zimbabwe**	864.96	2,239.95	3,104.91	918.25	945.63	1,863.88	1,241.04	0.13	0.34
**Total**	30,358.51	4,207,386.31	4,237,744.82	36,621.06	2,971,370.34	3,007,991.41	1,229,753.42		

Supplementary 16 table: Total DALYs averted and cost-effectiveness of implementing 2013 guidelines in a high cost less effective scenario

Country	Total DALY averted in 2013 guidelines (Thousands)	Total DALY averted in 2005 guidelines (Thousands)	Cost per DALY averted in 2013 guidelines (US$,2013)	Cost effectiveness comparing 2005 and 2013 guidelines (Cost per DALY saved, US$, 2013)	Percentage of cost per DALY saved in GDP per capita (%)
**Afghanistan**	457.30	875.44	70.58	21.72	3.51
**Azerbaijan**	23.97	45.64	281.51	223.39	3.02
**Bangladesh**	483.36	910.09	242.76	57.69	7.72
**China**	930.73	1,752.85	774.11	249.16	4.03
**Eritrea**	51.49	98.98	103.89	35.43	7.03
**Guatemala**	69.42	132.69	118.26	26.96	0.80
**India**	4,488.62	8,522.64	217.52	121.97	8.19
**Indonesia**	599.09	1,137.93	220.28	56.29	1.58
**Korea, Democratic People's Republic**	43.07	82.09	249.83	103.55	20.46
**Kyrgyzstan**	13.25	24.97	287.54	83.10	7.17
**Lao People's Democratic Republic**	64.96	124.61	74.67	27.82	1.99
**Madagascar**	193.75	370.93	86.90	22.64	5.06
**Mexico**	113.72	212.37	383.18	630.09	6.46
**Myanmar**	171.85	327.07	216.17	41.59	3.64
**Nepal**	86.92	165.78	270.76	34.58	4.89
**Niger**	330.60	635.69	50.91	13.41	3.50
**Pakistan**	1,670.98	3,212.46	81.51	24.80	1.92
**Papua New Guinea**	50.26	96.55	134.92	35.78	1.64
**Peru**	30.70	57.94	286.72	278.30	4.24
**Philippines**	314.84	595.98	189.49	103.01	3.98
**Senegal**	86.87	165.98	103.84	33.07	3.21
**Somalia**	247.51	476.59	32.16	10.99	0.15
**Tajikistan**	55.89	107.30	121.40	27.72	4.55
**Turkmenistan**	24.38	46.83	135.45	106.13	1.63
**Uzbekistan**	126.38	241.88	158.51	46.69	2.72
**Yemen**	159.17	303.25	129.50	67.28	4.50
**Bolivia (Plurinational State of)**	42.56	81.97	15.34	35.90	1.39
**Brazil**	91.80	167.92	91.73	293.56	2.59
**Cambodia**	62.68	120.25	14.82	24.79	2.62
**Comoros**	6.80	13.03	83.27	20.67	2.49
**Egypt**	119.66	227.57	37.99	102.26	3.21
**Iraq**	139.24	265.05	114.52	122.37	1.90
**Morocco**	79.31	151.78	84.10	67.12	2.31
**Solomon Islands**	2.34	4.47	51.09	46.23	3.05

Country	Total DALY averted in 2013 guidelines (Thousands)	Total DALY averted in 2005 guidelines (Thousands)	Cost per DALY averted in 2013 guidelines (US$,2013)	Cost effectiveness comparing 2005 and 2013 guidelines (Cost per DALY saved, US$, 2013)	Percentage of cost per DALY saved in GDP per capita (%)
**Viet Nam**	115.43	214.59	70.37	118.08	7.40
**Benin**	99.76	191.87	61.65	19.54	2.60
**Burkina Faso**	204.23	390.63	69.90	27.79	4.38
**Burundi**	129.84	250.15	72.89	7.12	2.84
**Cameroon**	259.10	499.15	47.30	19.21	1.67
**Congo**	19.80	37.44	125.20	127.33	4.04
**Congo, Democratic Republic**	204.74	1,770.31	234.16	6.90	2.54
**Ethiopia**	815.04	1,568.40	97.57	12.65	2.69
**Gambia**	15.50	29.69	61.40	23.55	4.60
**Ghana**	183.04	352.92	73.86	21.66	1.35
**Guinea**	116.81	224.05	73.20	18.66	3.16
**Liberia**	33.70	64.60	104.55	18.76	4.45
**Malawi**	112.32	215.99	131.22	12.53	4.67
**Mali**	267.58	515.92	44.88	14.20	2.05
**Mauritania**	42.13	80.87	68.36	24.23	2.19
**Mozambique**	221.78	423.41	107.73	18.59	3.21
**Nigeria**	2,335.38	4,499.06	53.31	20.18	1.30
**Rwanda**	90.65	174.96	107.74	11.60	1.87
**Sierra Leone**	95.41	184.08	43.04	11.34	1.79
**Togo**	60.93	117.10	77.32	17.45	0.39
**Uganda**	339.83	654.10	98.75	15.48	2.83
**Zambia**	161.14	311.04	65.01	14.49	0.99
**Angola**	502.54	971.25	13.52	39.31	0.72
**Botswana**	5.18	9.75	164.42	367.03	5.10
**Central African Republic**	59.19	113.76	137.89	-0.49	-0.10
**Chad**	268.10	516.30	8.00	15.23	1.72
**Côte d'Ivoire**	204.74	391.80	25.39	33.77	2.72
**Djibouti**	5.75	11.01	20.22	42.12	3.97
**Equatorial Guinea**	7.43	14.30	218.58	503.68	2.10
**Gabon**	8.57	16.33	89.78	269.99	2.36
**Guinea-Bissau**	21.72	41.79	9.45	15.43	2.86
**Haiti**	89.54	172.73	10.55	13.08	1.70
**Kenya**	424.58	817.99	20.55	14.59	1.69
**Lesotho**	13.00	24.95	25.52	22.34	1.87
**Sao Tome and Principe**	1.28	2.46	34.45	20.42	1.46
**South Africa**	168.74	320.65	119.66	206.96	13.10
**Sudan**	405.41	772.94	44.76	49.75	1.63
**Swaziland**	8.28	15.91	42.00	35.38	4.06
**Tanzania, United Republic of**	333.09	634.76	37.69	15.70	2.73
**Zimbabwe**	123.57	238.01	14.86	11.08	1.41
**Total**	20,722.69	204.50	145.23	59.27	
**Median**	112.32 (42.69	56.20 (34.69 –	83.27 (45.48 –	26.96	

Country	Total DALY averted in 2013 guidelines (Thousands)	Total DALY averted in 2005 guidelines (Thousands)	Cost per DALY averted in 2013 guidelines (US$,2013)	Cost effectiveness comparing 2005 and 2013 guidelines (Cost per DALY saved, US$, 2013)	Percentage of cost per DALY saved in GDP per capita (%)
**(IQR)**	-256.20)	120.61)	133.99)	(16.14-67.24)	

VI. Less effective scenario

Less effective scenario assumptions:

1. Mortality reduction with 2005 and 2013 guidelines: 36%

All other assumptions are the same as the main model.

Supplementary 17 table: Total DALYs averted and cost-effectiveness of implementing 2013 guidelines in a less effective scenario

Country	Total DALY averted (Thousands)	Cost per DALY averted (Thousands)		Percentage of cost per DALY saved in GDP per capita (%)	
		2005	2013	**2005**	**2013**
**Afghanistan**	457.30	74.66	58.16	12.05	9.39
**Azerbaijan**	23.97	404.56	206.97	5.47	2.80
**Bangladesh**	483.36	175.31	130.38	23.46	17.45
**China**	930.73	630.15	415.88	10.18	6.72
**Eritrea**	51.49	87.68	56.94	17.39	11.29
**Guatemala**	69.42	84.52	63.30	2.52	1.89
**India**	4,488.62	228.60	123.83	15.35	8.31
**Indonesia**	599.09	167.91	121.19	4.72	3.41
**Korea, Democratic People's Republic**	43.07	227.35	137.43	44.93	27.16
**Kyrgyzstan**	13.25	222.63	156.14	19.20	13.46
**Lao People's Democratic Republic**	64.96	64.52	41.15	4.61	2.94
**Madagascar**	193.75	66.78	49.03	14.93	10.96
**Mexico**	113.72	806.02	266.83	8.27	2.74
**Myanmar**	171.85	148.94	115.10	13.02	10.06
**Nepal**	86.92	172.48	145.25	24.41	20.55
**Niger**	330.60	43.51	33.17	11.36	8.66
**Pakistan**	1,670.98	71.93	51.27	5.57	3.97
**Papua New Guinea**	50.26	105.24	74.64	4.82	3.42
**Peru**	30.70	416.38	175.18	6.34	2.67
**Philippines**	314.84	200.01	113.27	7.73	4.38

Country	Total DALY averted (Thousands)	Cost per DALY averted (Thousands)		Percentage of cost per DALY saved in GDP per capita (%)	
		2005	2013	**2005**	**2013**
**Senegal**	86.87	86.29	59.12	8.36	5.73
**Somalia**	247.51	28.85	20.42	0.38	0.27
**Tajikistan**	55.89	98.13	75.54	16.12	12.41
**Turkmenistan**	24.38	176.60	81.70	2.71	1.25
**Uzbekistan**	126.38	128.61	89.13	7.49	5.19
**Yemen**	159.17	141.21	85.13	9.45	5.70
**Bolivia (Plurinational State of)**	42.56	45.87	14.44	1.78	0.56
**Brazil**	91.80	317.81	85.99	2.80	0.76
**Cambodia**	62.68	33.86	13.53	3.58	1.43
**Comoros**	6.80	97.28	81.65	11.71	9.83
**Egypt**	119.66	123.68	35.84	3.88	1.12
**Iraq**	139.24	218.14	112.12	3.38	1.74
**Morocco**	79.31	140.76	82.55	4.85	2.84
**Solomon Islands**	2.34	87.64	49.25	5.77	3.24
**Viet Nam**	115.43	159.00	66.08	9.96	4.14
**Benin**	99.76	49.35	33.32	6.56	4.43
**Burkina Faso**	204.23	63.14	41.13	9.95	6.48
**Burundi**	129.84	48.00	42.65	19.13	16.99
**Cameroon**	259.10	42.82	26.56	3.72	2.31
**Congo**	19.80	192.49	83.78	6.10	2.66
**Congo, Democratic Republic**	204.74	172.60	128.54	63.46	47.26
**Ethiopia**	815.04	60.74	50.77	12.92	10.80
**Gambia**	15.50	55.47	36.61	10.83	7.15
**Ghana**	183.04	58.94	40.47	3.67	2.52
**Guinea**	116.81	57.75	43.06	9.77	7.29
**Liberia**	33.70	73.70	59.37	17.48	14.08
**Malawi**	112.32	83.85	74.15	31.28	27.66
**Mali**	267.58	40.16	28.76	5.79	4.14
**Mauritania**	42.13	65.48	45.53	5.92	4.12
**Mozambique**	221.78	92.83	78.82	16.04	13.62
**Nigeria**	2,335.38	54.80	37.76	3.52	2.43
**Rwanda**	90.65	67.45	58.12	10.88	9.38
**Sierra Leone**	95.41	33.34	24.40	5.25	3.84
**Togo**	60.93	58.07	44.19	1.29	0.98
**Uganda**	339.83	66.08	53.80	12.08	9.84
**Zambia**	161.14	50.70	38.50	3.45	2.62
**Angola**	502.54	48.54	12.91	0.88	0.24
**Botswana**	5.18	478.19	159.13	6.65	2.21
**Central African Republic**	59.19	133.60	136.21	28.26	28.82
**Chad**	268.10	19.47	6.97	2.20	0.79
**Côte d'Ivoire**	204.74	51.18	23.37	4.11	1.88
**Djibouti**	5.75	53.39	18.25	5.03	1.72
**Equatorial Guinea**	7.43	680.42	216.78	2.83	0.90
**Gabon**	8.57	329.20	87.69	2.88	0.77
**Guinea-Bissau**	21.72	20.49	8.07	3.80	1.50
**Haiti**	89.54	20.29	9.66	2.63	1.25

Country	Total DALY averted (Thousands)	Cost per DALY averted (Thousands)		Percentage of cost per DALY saved in GDP per capita (%)	
		2005	2013	**2005**	**2013**
**Kenya**	424.58	31.10	19.27	3.60	2.23
**Lesotho**	13.00	40.20	21.71	3.37	1.82
**Sao Tome and Principe**	1.28	50.44	33.41	3.60	2.38
**South Africa**	168.74	298.27	115.40	18.88	7.30
**Sudan**	405.41	83.26	42.55	2.74	1.40
**Swaziland**	8.28	70.13	38.98	8.04	4.47
**Tanzania, United Republic of**	333.09	47.81	35.92	8.33	6.26
**Zimbabwe**	123.57	21.90	12.93	2.78	1.64
**Total**	20,722.69	141.85	85.83	12.05	9.39
**Median**	112.32				
**(IQR)**	(42.69-256.20)	74.66(50.51-171.34)	53.80 (35.86-88.77)		

VII. Not cost-effective scenario

High cost less effective scenario assumptions:

4. CCM Coverage: 100% coverage of rural population in countries with CCM
5. Unit cost of medicine changed based on review by Zhang et al 2015 (in press)
6. Mortality reduction with 2013 guidelines: 9%, 2005 guidelines remain 70%.

All other assumptions are the same as the main model.

Supplementary 18 table: Total DALYs averted and cost-effectiveness of implementing 2013 guidelines in not cost-effective scenario

Country	Total DALY averted in 2013 guidelines (Thousands)	Total DALY averted in 2005 guidelines (Thousands)	Cost per DALY averted in 2013 guidelines (US$, 2013)	Percentage of cost per DALY saved in 2013 in GDP per capita (%)	Cost effectiveness ratio comparing 2005 and 2013 guidelines (Cost per DALY saved, US$, 2013)	Percentage of Cost-effectiveness ratio comparing 2005 and 2013 in GDP per capita (%)
**Afghanistan**	125.25	875.44	257.70	41.59	12.11	1.95
**Azerbaijan**	6.75	45.64	998.83	13.51	124.51	1.68
**Bangladesh**	144.49	910.09	812.11	108.67	32.16	4.30
**China**	277.87	1,752.85	2592.86	41.90	138.88	2.24
**Eritrea**	13.78	98.98	388.33	77.00	19.75	3.92
**Guatemala**	19.18	132.69	428.07	12.77	15.03	0.45
**India**	1,285.13	8,522.64	759.73	51.01	67.98	4.56
**Indonesia**	171.19	1,137.93	770.89	21.67	31.38	0.88
**Korea, Democratic People's**	12.09	82.09	890.20	175.93	57.72	11.41

Country	Total DALY averted in 2013 guidelines (Thousands)	Total DALY averted in 2005 guidelines (Thousands)	Cost per DALY averted in 2013 guidelines (US$, 2013)	Percentage of cost per DALY saved in 2013 in GDP per capita (%)	Cost effectiveness ratio comparing 2005 and 2013 guidelines (Cost per DALY saved, US$, 2013)	Percentage of Cost-effectiveness ratio comparing 2005 and 2013 in GDP per capita (%)
**Republic Kyrgyzstan**	3.95	24.97	965.38	83.25	46.32	3.99
**Lao People's Democratic Republic**	17.60	124.61	275.58	19.70	15.50	1.11
**Madagascar**	53.04	370.93	317.41	70.94	12.62	2.82
**Mexico**	35.37	212.37	1231.86	12.64	351.20	3.60
**Myanmar**	48.59	327.07	764.58	66.83	23.18	2.03
**Nepal**	24.30	165.78	968.61	137.07	19.28	2.73
**Niger**	88.31	635.69	190.57	49.78	7.48	1.95
**Pakistan**	446.87	3,212.46	304.78	23.62	13.83	1.07
**Papua New Guinea**	13.50	96.55	502.19	22.99	19.95	0.91
**Peru**	9.07	57.94	970.67	14.78	155.12	2.36
**Philippines**	91.58	595.98	651.44	25.18	57.42	2.22
**Senegal**	24.05	165.98	375.09	36.36	18.43	1.79
**Somalia**	65.60	476.59	121.36	1.62	6.13	0.08
**Tajikistan**	15.07	107.30	450.21	73.94	15.45	2.54
**Turkmenistan**	6.55	46.83	504.43	7.75	59.16	0.91
**Uzbekistan**	34.65	241.88	578.05	33.68	26.02	1.52
**Yemen**	44.75	303.25	460.59	30.82	37.50	2.51
**Bolivia (Plurinational State of)**	11.26	81.97	57.99	2.25	20.01	0.78
**Brazil**	31.34	167.92	268.66	2.37	163.63	1.44
**Cambodia**	16.96	120.25	54.75	5.79	13.82	1.46
**Comoros**	1.86	13.03	304.42	36.65	11.52	1.39
**Egypt**	33.98	227.57	133.79	4.20	57.00	1.79
**Iraq**	39.33	265.05	405.45	6.28	68.20	1.06
**Morocco**	21.75	151.78	306.63	10.57	37.41	1.29
**Solomon Islands**	0.65	4.47	183.48	12.09	25.77	1.70
**Viet Nam**	36.68	214.59	221.43	13.88	65.82	4.12
**Benin**	26.61	191.87	231.12	30.74	10.89	1.45
**Burkina Faso**	56.20	390.63	254.01	40.04	15.49	2.44
**Burundi**	34.29	250.15	275.97	109.96	3.97	1.58
**Cameroon**	68.47	499.15	178.99	15.55	10.71	0.93
**Congo**	5.79	37.44	428.26	13.58	70.97	2.25
**Congo, Democratic Republic**	56.20	1,770.31	853.09	**313.67**	6.30	2.32
**Ethiopia**	216.79	1,568.40	366.83	78.01	7.05	1.50
**Gambia**	4.23	29.69	224.89	43.92	13.12	2.56
**Ghana**	48.14	352.92	280.84	17.50	12.07	0.75
**Guinea**	31.65	224.05	270.16	45.71	10.40	1.76
**Liberia**	9.16	64.60	384.51	91.18	10.46	2.48
**Malawi**	30.00	215.99	491.33	183.29	6.98	2.61

Country	Total DALY averted in 2013 guidelines (Thousands)	Total DALY averted in 2005 guidelines (Thousands)	Cost per DALY averted in 2013 guidelines (US$, 2013)	Percentage of cost per DALY saved in 2013 in GDP per capita (%)	Cost effectiveness ratio comparing 2005 and 2013 guidelines (Cost per DALY saved, US$, 2013)	Percentage of Cost-effectiveness ratio comparing 2005 and 2013 in GDP per capita (%)
**Mali**	70.36	515.92	170.66	24.59	7.91	1.14
**Mauritania**	11.37	80.87	253.28	22.90	13.51	1.22
**Mozambique**	61.65	423.41	387.51	66.95	10.36	1.79
**Nigeria**	617.17	4,499.06	201.72	12.97	11.25	0.72
**Rwanda**	23.70	174.96	412.09	66.47	6.47	1.04
**Sierra Leone**	24.99	184.08	164.31	25.88	6.32	1.00
**Togo**	16.31	117.10	288.78	6.43	9.73	0.22
**Uganda**	90.26	654.10	371.78	67.97	8.63	1.58
**Zambia**	42.10	311.04	248.82	16.94	8.07	0.55
**Angola**	130.32	971.25	52.14	0.95	21.91	0.40
**Botswana**	1.54	9.75	551.60	7.67	204.58	2.84
**Central African Republic**	15.86	113.76	514.68	108.89	-0.27	-0.06
**Chad**	70.99	516.30	30.22	3.41	8.49	0.96
**Côte d'Ivoire**	56.20	391.80	92.49	7.44	18.83	1.51
**Djibouti**	1.58	11.01	73.65	6.94	23.47	2.21
**Equatorial Guinea**	1.98	14.30	821.44	3.42	280.74	1.17
**Gabon**	2.40	16.33	319.89	2.80	150.49	1.32
**Guinea-Bissau**	5.78	41.79	35.50	6.58	8.60	1.59
**Haiti**	23.48	172.73	40.21	5.22	7.29	0.95
**Kenya**	112.17	817.99	77.78	8.99	8.13	0.94
**Lesotho**	3.51	24.95	94.57	7.93	12.45	1.04
**Sao Tome and Principe**	0.34	2.46	128.59	9.17	11.38	0.81
**South Africa**	48.10	320.65	419.76	26.57	115.35	7.30
**Sudan**	113.54	772.94	159.81	5.25	27.73	0.91
**Swaziland**	2.21	15.91	157.30	18.03	19.72	2.26
**Tanzania, United Republic of**	93.54	634.76	134.21	23.38	8.75	1.52
**Zimbabwe**	32.69	238.01	56.18	7.13	6.18	0.78
**Total**	11,431.00	204.50	263.27	41.59	107.45	
**Median (IQR)**	31.34 (11.55 -64.61)	56.20 (34.69 – 120.61)	304.42 (172.74 – 499.48)		15.45(9.88-37.48)	

Supplementary 19 table: Years of Life Saved and deaths averted in each country by implementing 2013 guidelines

Country	Number of deaths averted	Years of Life Saved (Years)

Country	Number of deaths averted	Years of Life Saved (Years)
**Afghanistan**	14,065.1	860,870.2
**Angola**	18,503.8	965,004.0
**Azerbaijan**	629.3	44,624.6
**Bangladesh**	12,378.8	878,558.1
**Benin**	3,179.4	189,644.0
**Bolivia**	1,201.2	81,147.9
**Botswana**	197.4	9,418.2
**Brazil**	2,111.2	156,735.0
**Burkina Faso**	6,786.5	383,766.7
**Burundi**	4,552.8	247,700.2
**Cambodia**	1,647.8	118,524.1
**Cameroon**	8,929.9	494,226.7
**Central African Republic**	2,228.8	112,342.2
**Chad**	9,933.0	511,019.4
**China**	22,367.8	1,692,596.7
**Comoros**	209.3	12,815.8
**Congo**	614.6	36,324.7
**Dem. Rep. of the Congo**	34,817.3	1,752,467.8
**Côte d'Ivoire**	7,546.0	385,116.8
**Djibouti**	174.3	10,822.6
**Egypt**	3,106.6	222,153.1
**Equatorial Guinea**	264.6	14,138.9
**Eritrea**	1,552.6	97,777.8
**Ethiopia**	24,313.8	1,551,032.8
**Gabon**	250.6	15,976.7
**Gambia**	493.5	29,218.0
**Ghana**	5,693.1	349,750.9
**Guatemala**	1,800.4	130,257.2
**Guinea**	3,915.8	220,789.8
**Guinea-Bissau**	756.0	41,323.6
**Haiti**	2,701.3	171,264.2
**India**	124,437.6	8,305,330.8
**Indonesia**	15,588.3	1,109,376.8
**Iraq**	3,703.7	259,019.7
**Kenya**	13,056.4	809,952.7
**Dem. Peoples's Rep. of Korea**	1,144.5	80,329.8
**Kyrgyzstan**	354.9	24,123.3
**Lao People's Dem. Republic**	1,792.7	122,790.4
**Lesotho**	493.5	24,607.4
**Liberia**	1,047.9	63,612.2
**Madagascar**	5,614.7	364,793.5
**Malawi**	3,845.8	213,434.1
**Mali**	9,250.5	511,294.9
**Mauritania**	1,288.0	79,755.0
**Mexico**	2,613.8	203,115.4
**Morocco**	2,094.4	149,216.4
**Mozambique**	8,202.6	415,132.1
**Myanmar**	4,878.3	319,571.7
**Nepal**	2,364.6	162,357.7
**Niger**	10,710.7	628,135.7
**Nigeria**	84,436.1	4,454,629.1
**Pakistan**	47,395.6	3,173,625.6
**Papua New Guinea**	1,518.3	95,295.4
**Peru**	746.2	56,080.2

Country	Number of deaths averted	Years of Life Saved (Years)
**Philippines**	8,374.8	578,819.4
**Rwanda**	2,709.7	173,580.6
**Sao Tome and Principe**	36.4	2,426.6
**Senegal**	2,557.8	162,869.1
**Sierra Leone**	3,985.1	182,558.4
**Solomon Islands**	64.4	4,381.7
**Somalia**	8,519.7	471,631.5
**South Africa**	5,445.3	312,757.2
**Sudan**	12,126.1	756,689.6
**Swaziland**	317.1	15,726.3
**Tajikistan**	1,562.4	105,836.0
**United Republic of Tanzania**	10,047.8	621,073.3
**Togo**	2,034.2	115,661.8
**Turkmenistan**	701.4	46,228.9
**Uganda**	10,874.5	647,026.4
**Uzbekistan**	3,462.9	237,798.7
**Viet Nam**	2,684.5	204,153.3
**Yemen**	4,673.2	296,641.3
**Zambia**	5,306.7	308,620.2
**Zimbabwe**	3,911.6	235,614.4
**Total**	632,897.3	38,893,083.7

Suppementary Box 1 Key changes in the classification of pneumonia and recommended treatment in the 2005 guideline and 2013 guideline

2005 Guidelines				2013 Guidelines		
**Patient Group**	**Classification of Pneumonia**	**Major Symptoms**	**Recommended Treatment**	**Classification of Pneumonia**	**Major Symptoms**	**Recommended Treatment**
**HIV-**	Pneumonia	Fast breathing	5 days treatment at community or first level health facility with co-trimoxazole (non-dispersible tablet at recommended dose)	Pneumonia	Fast breathing	5 days treatment at community or first level health facility with oral amoxicillin (dispersible tablet at recommended dose)
	Severe pneumonia	Chest indrawing	Admit to hospital		Chest indrawing	5 days treatment at first level health facility
	Very severe pneumonia	Danger signs	Admit to hospital	Severe pneumonia	Danger signs	Admit to hospital
**HIV+**	Pneumonia	Fast breathing	5 days treatment at community or first level health facility with co-trimoxazole (non-dispersible	Pneumonia	Fast breathing	5 days treatment at community or first level health facility with oral amoxicillin (dispersible

		tablet at recommended dose)			tablet at recommended dose)
Severe pneumonia	Chest indrawing	Admit to hospital		Chest indrawing	Admit to hospital
Very severe pneumonia	Danger signs	Admit to hospital	Severe pneumonia	Danger signs	Admit to hospital

Suppementary Box 2 Description of scenarios

Scenarios	New Assumptions
Baseline scenario	1. Universal coverage: 100% patients get treatment at community, fist level facility or hospital.<br>2. CCM Coverage: 50% rural population would be treated by CHWs and the remaining 50% would be treated at a health facility. [15,29]<br>3. The proportion of pneumonia signs (i.e. fast breathing, lower chest wall indrawing and danger signs) are the same in HIV-infected and uninfected children with pneumonia – 85% of children with pneumonia have fast breathing, 13% have chest wall indrawing and about 2% have danger signs – based on the results of studies carried out at the community level. [15,22,23]<br>4. The number of CHWs needed per country: one CHW per 1000 rural population.[29]<br>5. Deaths averted by pneumonia treatment were calculated based on the reported estimates of 70% child mortality reduction from universal coverage of community management of childhood pneumonia [34]<br>6. Unit cost of medicine : We used median supplier prices from Management Sciences for Health (MSH) International Price Indicator (2012)[30], UNICEF supply division data, and UNICEF report for cost of medicines[31] for the list of drugs based on the WHO guidelines
High cost scenario	1. CCM Coverage: 100% coverage of rural population in countries with CCM<br><br>2. Unit cost of medicine changed based on review by Zhang et al 2016 [19]
Low cost scenario	1. CCM Coverage: coverage reduced to 50% coverage of rural population in countries with CCM, remaining 50% were treated at health centre.<br><br>2.One Community Health Worker per 5000 rural population
Effective access scenario	Based on data from published studies[39] (Guenther et al. 2012) reporting effective access to care (product of geographic access, staff availability and medicine availability)<br><br>1. In countries without CCM in place, effective access rate for rural population is 9%, the remaining 91% of rural population will not have access to care<br><br>2. In countries with CCM in place, effective access rate for rural population is 30% in a typical setting, the remaining 70% of rural population will not have access to care

New (unpublished) data for proportion of pneumonia signs in HIV+ children	In high HIV prevalence setting (Malawi) the proportion of pneumonia cases with fast breathing only/ chest indrawing/ dangerous signs has been reported 85%, 1.5%, 13.5% (Tim Colbourn, personal communication). These are program data reported by health workers trained in iCCM. We applied this split to the estimated number of HIV-infected children with pneumonia reported in the literature. Split for HIV uninfected children remained as before- i.e. 85%, 13%, 2%.
High cost less effective scenario	1. CCM Coverage: 100% coverage of rural population in countries with CCM<br><br>2. Unit cost of medicine changed based on review by Zhang et al 2016 [19]<br><br>3. Mortality reduction with 2013 guideline: 36% (Theodoratou et al, 2010)[34], 2005 guideline remains 70%.
